# Do France, Germany, and Italy agree on the added therapeutic value of medicines?

**DOI:** 10.1017/S026646232300048X

**Published:** 2023-08-15

**Authors:** Giorgio Casilli, Dario Lidonnici, Claudio Jommi, Marika De Nigris, Armando A. Genazzani

**Affiliations:** 1 More Than Access (MTA) Srl SB, Milan, Italy; 2 Department of Pharmaceutical Sciences, Università del Piemonte Orientale “Amedeo Avogadro”, Novara, Italy; 3 Pharmalex Italy SpA, Milan, Italy

**Keywords:** added therapeutic value, HTA organizations, health technology assessment, innovativeness, Joint Clinical Assessment

## Abstract

**Objectives:**

The Health Technology Assessment (HTA) of medicines is performed separately at the country level with some differences, but Italy, France, and Germany have implemented price and reimbursement systems strongly focused on the Added Therapeutic Value (ATV). This study investigates the level of agreement on ATV assessments by Agenzia Italiana del Farmaco (AIFA), Haute Autorité de Santé (HAS), and Gemeinsamer Bundesausschuss (G-BA).

**Methods:**

A database was created collecting all information about drugs with innovativeness status requests in Italy from July 2017 to December 2022 and populated with the corresponding HAS and G-BA ATV assessments. The primary comparative analysis was conducted by grouping the ATV ratings into “higher added value” and “lower or no added value”, while a secondary analysis considered the Italian innovativeness status as a criterion to include the quality of evidence assessment. The concordance between ATV assessments was investigated through percentage agreement and unweighted Cohen k-value.

**Results:**

189 medicines/indications were included. The greatest agreement was found when comparing G-BA versus HAS (82 percent; k = 0.61, substantial agreement). Lower levels of agreements were observed for AIFA versus HAS and AIFA versus G-BA (respectively 52 percent; k = 0.17 and 57 percent; k = 0.25). The secondary analysis led to a reconciliation to moderate agreement for AIFA versus HAS (72 percent; k = 0.45) and AIFA versus G-BA (74 percent; k = 0.47).

**Conclusions:**

A high degree of concordance between HTA organizations is reached when considering jointly ATV and quality of evidence, suggesting that the system is extensively mature to make a Joint Clinical Assessment, avoiding duplications and reducing access inequalities.

## Background

Once the European Medicines Agency (EMA) approves new medicinal products based on their absolute benefit–risk, the drug will be available in all countries of the European Union. Yet, whether it will be reimbursed will depend on assessments and appraisals done at the national or subnational levels and on price and reimbursement negotiations (1).

National parallel assessments allow each Member State to evaluate the technologies considering local needs, available alternatives, and organizational issues. However, parallel value assessments of new medicinal products can lead to a disparity in patient access to treatments across Europe, and inefficiencies in the management of HTA.

The new European HTA regulation (2), and the Joint Clinical Assessment (JCA) in particular, aims at facing the challenges posed by national parallel assessment, that is unequal market access and duplication of work for national HTA organizations.

Our aim is scrutinizing the level of concordance of the present assessment and appraisal of the added therapeutic value by HTA organizations in the three largest European markets (France, Germany, and Italy). A high level of concordance would make the JCA easier to implement.

In fact, these countries have national HTA organizations that evaluate new drugs defining their place in therapy and assessing their (added) value (3).

In France, the Transparency Commission (Commission de la Transparence, CT) of the Haute Autorité de Santé (HAS) deals with the scientific evaluation of medicinal products for reimbursement and pricing purposes (4). Specifically, the CT evaluates and ranks in four levels new drugs according to the absolute benefit of the drug (Service Médical Rendu, SMR), and in five levels (from a major additional benefit to no added benefit) the added therapeutic value of the new drug compared to the current medical practice and the French standard of care (Amélioration du Service Médical Rendu, ASMR). The SMR is used to decide the reimbursement status (the decision is taken by the Social Insurance system), while the ASMR is used, among other parameters by the Pricing Committee (Comité Économique des Produits de Santé, CEPS), to negotiate prices: only medicines with substantial additional benefit can aspire to a price premium (5). The decisions regarding SMR and ASMR are then made publicly available (6).

In Germany, companies are free to set prices at market launch. However, since 2011, they must submit a dossier to the Federal Joint Committee (Gemeinsame Bundesausschuss, G-BA) to allow them to define the Clinical Added Benefit (CAB) of the new medicinal product over an appropriate comparator decided by the G-BA. The dossier is evaluated in most cases by the Institute for Quality and Efficiency in Health Care (Institut für Qualität und Wirtschaftlichkeit im Gesundheitswesen, IQWiG), which provides an early benefit assessment (7;8) (publicly available on its website (9)). This early assessment drives a recommendation by the G-BA, which will make the final decision, sometimes diverging from IQWiG (10). The CAB is ranked in six levels (from major added benefit to lesser benefit), and a discount over the list price is negotiated with the association of the social insurances within one year of market launch. Also, the G-BA makes its final assessments and decisions public (8).

In Italy, both the assessment and the negotiation of price and reimbursement are managed by the Italian Medicines Agency (Agenzia Italiana del Farmaco, AIFA). AIFA is supported by two committees: the Scientific Committee (Commissione Tecnico-Scientifica, CTS) and the Price and Reimbursement Committee (Comitato Prezzi e Rimborso, CPR). Marketing authorization holders may apply for any new indication to be recognized as innovative, that is, a tag that allows the indication to claim some access benefits: a dedicated fund that covers the expenditure in the first three years and immediate access to the regional markets. Evaluation of innovativeness is based on three criteria: the therapeutic need (based on the value of the alternatives), the added therapeutic value (ATV), and the quality of the evidence provided (11;12). The final evaluation for those medicines that claim innovative status, unlike that of all the other drugs, is made publicly available (13). The unmet need/ATV and the quality of the evidence are ranked through a five-level and four-level scale. The CTS can award a full (all benefits are recognized) or potential innovativeness status (only immediate access to regional markets is granted). The evaluation of the quality of the evidence is managed separately from the ATV, differently from the other countries where it is embedded into the therapeutic value judgment. While these three dimensions are all considered to draw a final conclusion, they do not enter in a strict algorithm but are weighed on a case-by-case basis. Furthermore, there is no formal link between innovativeness status and price and reimbursement negotiation.

For the subset of drugs for which the innovativeness status has been requested to AIFA, the overall conclusion of the three countries can be compared from publicly available sources.

In the current work, we present the level of agreement of the three agencies for all drugs that have requested the innovativeness status in Italy from July 2017 to December 2022. To our knowledge, this is the first and most up-to-date study comparing assessments among the top three European Health Technology Assessment (HTA) organizations, although recent analyses have made comparisons between two of these HTA agencies: AIFA versus HAS (14); HAS versus G-BA (7); HAS versus IQWiG (15).

## Methods

A database was created starting from the innovation reports available on the AIFA website (16). The innovativeness reports are available once the price and reimbursement decisions are finalized and published in the Italian Official Gazette. The data cut-off was December 2022. These reports list AIFA’s assessment of the three criteria for innovative status recognition (therapeutic need, ATV, quality of evidence) for each single indication from July 2017 (start date of report publications) to December 2022 (latest data available before processing). From the indications included in these reports, the database was then populated by extracting from HAS (4) and G-BA (17) websites the corresponding ASMR and CAB assessments (data cut-off December 2022). 189 medicinal products/therapeutic indications were thereby included. It should be noted that in some cases AIFA and HAS apply a restriction on the reimbursable population and consequently on its ATV assessment; therefore, the therapeutic indications evaluated are not always perfectly overlapping. Moreover, compared to AIFA and HAS, the G-BA often performs the CAB assessment based on patient subgroups, and hence more than one CAB evaluation was present. In these cases, a critical case-by-case evaluation of the CAB assessments in the various subpopulations was performed by considering the assessment in the subgroup closest to the specific therapeutic indication evaluated by AIFA for innovative status recognition. In case of multiple and conflicting ratings, these drugs/indications were excluded from the analysis. This approach allowed HTA assessments for the same (or at least the most similar) therapeutic indication to be analyzed in an attempt to minimize possible biases in interpreting the assessments.

**Figure 1.**
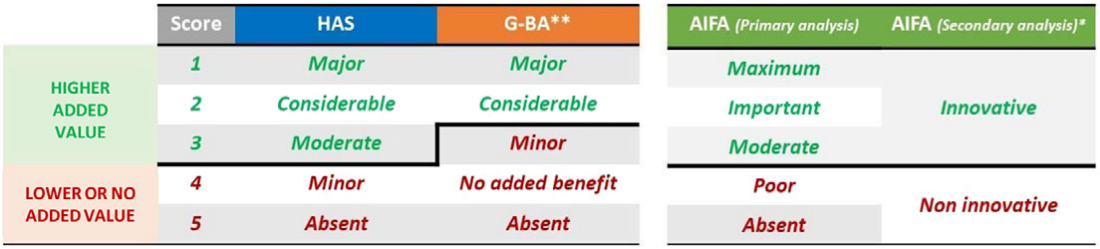
Grouping in “higher added value” and “lower or no added value” based on ATV, ASMR, and CAB levels of each HTA agency. (*Conditionally innovative drugs were excluded from the analysis; **G-BA’s “non-quantifiable” rating was excluded since not clearly classifiable in comparison with AIFA and HAS ratings; AIFA indicates Agenzia Italiana del Farmaco; ASMR, Amélioration du Service Médical Rendu; ATV, Added Therapeutic Value; CAB, Clinical Added Benefit; G-BA, Gemeinsame Bundesausschuss; HAS, Haute Autorité de Santé; HTA, Health Technology Assessment).

All three agencies define the ATV on a scale, although such scale slightly differs in terms of the number of scores and definitions (Supplementary Table 1 (18–20)). Analyzing the definition of ATV levels assigned by each HTA organization, the analysis was conducted by grouping the ratings into “higher added value” and “lower or no added value” as shown in Figure 1. Since the G-BA’s “non-quantifiable” assessment rating is not clearly classifiable in the “lower or no added value” group, it was deemed appropriate not to consider the medicinal products/indications with this rating in the analysis.

As mentioned before, AIFA provides for an innovativeness judgment on the grounds of two criteria besides the ATV: unmet need and quality of the evidence. The former has a limited impact on the actual appraisals and the latter is embedded into the ATV appraisal in other countries. Therefore, we carried out a secondary analysis using the innovative status itself as a criterion to classify the appraisals into “higher added value” and “lower or no added value”.

Subgroup analyses were conducted according to orphan designated indications and first level of Anatomical Therapeutic Chemical Classification System (ATC) “L”- antineoplastic and immunomodulating agents (being the most represented ATC code).

An alternative analysis was conducted grouping the ATV assessments into four categories (Major, Important, Minor, and No proof of benefit) to evaluate the concordance between the three agencies (see Supplementary Table 2 for more details). The classification is inspired by a previous work done by Boucaud-Maitre et al. (15). In the cross-HTA organization comparison, medicinal products/therapeutic indications were excluded for the following reasons: (a) for fourteen indications, AIFA assessment was “non-assessable”; (b) for twenty indications, HAS assessment on ASMR was missing; (c) for one indication, there were multiple and conflicting ratings from HAS; (d) for twenty-one indications, G-BA assessment on CAB was missing; (e) for eighteen indications, there were multiple and conflicting ratings from G-BA assessment; (f) for thirty-seven indications, the final CAB assessment of the G-BA was “non-quantifiable”. In conclusion, the analysis of the ATV assessment concordance between AIFA and HAS was conducted on 159 medicinal products/therapeutic indications, 101 for AIFA-G-BA comparison, and 101 for that between HAS and G-BA.

### Statistical analysis

Descriptive analyses of the sample were conducted, expressing the frequency and percentage of quantitative data. Contingency tables were used to represent and analyze the relationships between inter-agency ATV scores: the columns represented the different raters (HTA agencies), and the rows represented variables for which the raters have made the assessment (higher added value/lower or no added value).

To investigate concordance between HTA evaluations on the ATV, percentage agreement and unweighted Cohen k-values were used (21;22).

## Results

### Description of the sample and general level of agreement

Overall, 189 medicinal products/indications were analyzed (Supplementary Table 3), of which 87 (46 percent) were orphan and 118 (62 percent) were antineoplastic and immunomodulating agents (ATC code: L). Sixty-three (33 percent) indications were recognized as innovative by AIFA and fifty-one (27 percent) were assessed as conditionally innovative.

**Figure 2.**
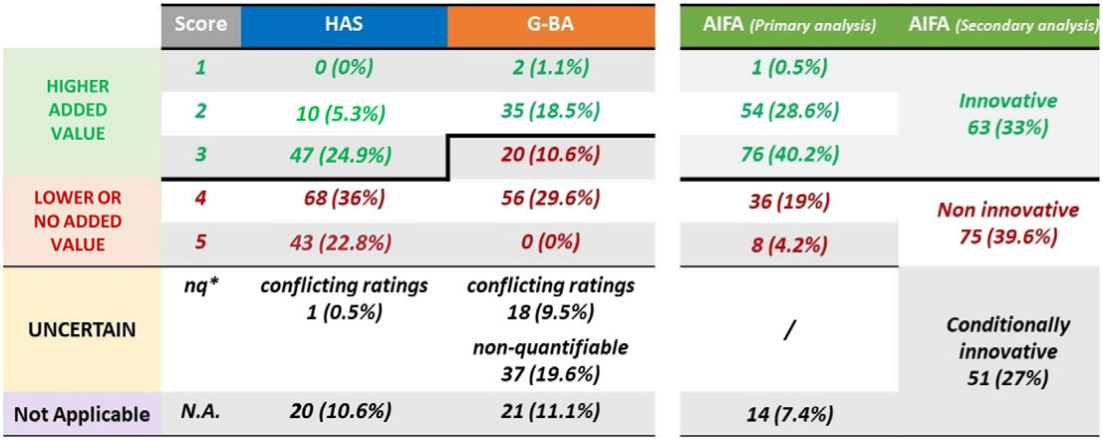
Evaluation of the added therapeutic value of AIFA, HAS, and G-BA (*nq = non-quantifiable; AIFA indicates Agenzia Italiana del Farmaco; G-BA, Gemeinsame Bundesausschuss; HAS, Haute Autorité de Santé).

Figure 2 shows the evaluation of the added therapeutic value by the three agencies. AIFA appears to be the most lenient evaluator, while HAS appears to be the strictest HTA organization. Overall, AIFA classified 69 percent of drugs as having higher added value. On the contrary, HAS classified 30.2 percent and G-BA classified 19.6 percent of drugs in the “higher added value” category.

**Figure 3.**
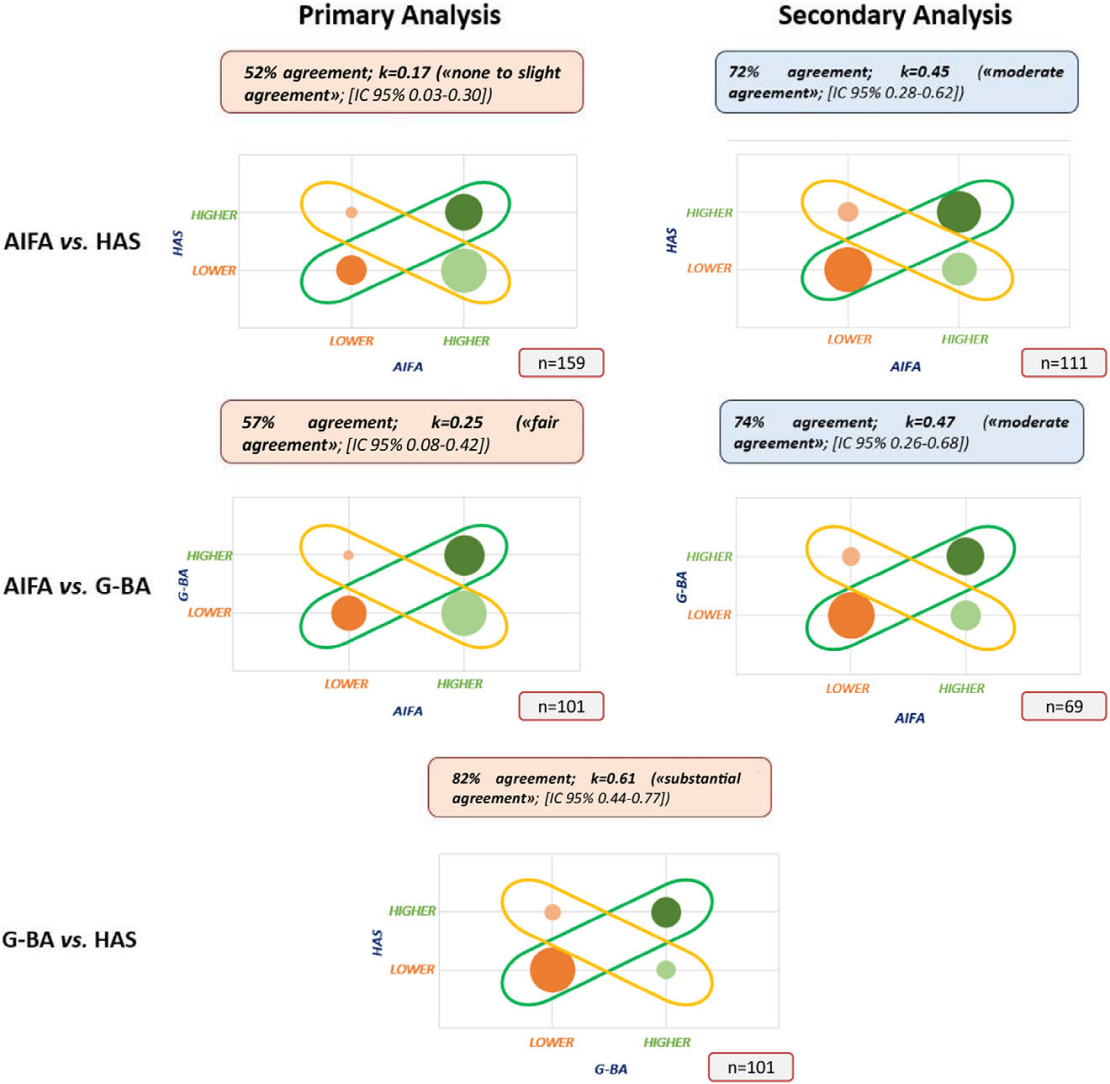
Concordance on ATV assessments among HTA bodies (Note: the size of the bubbles represents the sample size; AIFA indicates Agenzia Italiana del Farmaco; ATV, Added Therapeutic Value; CAB, Clinical Added Benefit; G-BA, Gemeinsame Bundesausschuss; HAS, Haute Autorité de Santé; HTA, Health Technology Assessment).

We then analyzed the concordance of the decisions made by the three agencies (Figure 3). The greatest level of inter-agency agreement was found when comparing the G-BA and HAS assessments of the added value. An agreement rate of 82 percent was found (n = 101), with Cohen k-value equal to 0.61 [CI 95 percent 0.44–0.77] (Figure 3), showing that there is a substantial agreement as per definition. A similar level of agreement was confirmed for ATC code “L” subgroup analysis (n = 67): percentage agreement 85 percent; k = 0.66 (substantial agreement; [CI 95 percent 0.46–0.85]). On the contrary, the percentage of agreement decreases for orphan indications (n = 39): percentage agreement 69 percent; k = 0.34 (fair agreement; [CI 95 percent 0.02–0.65]).

When analyzing the relationship between AIFA and the other two agencies using the ATV of AIFA (primary analysis), instead, strong divergences emerged. The agreement between AIFA and HAS (n = 159 indications) fell to 52 percent with a Cohen k-value of 0.17 (none to slight agreement; [CI 95 percent 0.03–0.30]), while the agreement between AIFA and G-BA (n = 101 indications) was 57 percent, with a Cohen k-value of 0.25 (fair agreement; [CI 95 percent 0.08–0.42]). Subgroup analyses returned a similar picture both for the “L” ATC code (AIFA versus HAS: n = 98; 47 percent agreement; k = 0.13 [CI 95 percent 0–0.29]; AIFA versus G-BA: n = 65; 60 percent agreement; k = 0.30 [CI 95 percent 0.08–0.50]) or for orphan products (AIFA versus HAS: n = 76; 47 percent agreement; k = 0.10 [CI 95 percent 0–0.29]; AIFA versus G-BA: n = 40; 60 percent agreement; with k = 0.29 [CI 95 percent 0.01–0.55]) (Supplementary Figure 1).

As mentioned above, AIFA defines innovativeness by combining the ATV with two further dimensions: the therapeutic need, which has been shown previously not to be a driver of decisions (11;12) and the quality of evidence. We therefore performed a secondary analysis using the combined decision instead of the only ATV assessment, that is focusing only on medicines/indications that were appraised as fully innovative or non-innovative according to AIFA. This analysis led to a reconciliation of the assessments with 72 percent agreement between AIFA and HAS (k = 0.45; moderate agreement; [CI 95 percent 0.28–0.62]) and 74 percent agreement between AIFA and G-BA (k = 0.47; moderate agreement; [CI 95 percent 0.26–0.68]).

A more granular, but less robust from a statistical standpoint, supplementary analysis was performed as it was done by Boucaud-Maitre et al. (15), without grouping the assessments into two clusters. This analysis substantially reduces the level of agreement between G-BA and HAS equal to 50 percent (k = 0.30; [CI 95 percent 0.16–0.43]), which, however, remains well above the ones between AIFA and the other two HTA organizations (AIFA versus HAS: 46 percent agreement; AIFA versus G-BA: 45 percent agreement) (see Supplementary Table 2 for more details).

## Discussion

The present contribution aims at evaluating the current concordance between the assessment of the ATV of medicines in the three largest European pharmaceutical markets, in view of the new European HTA regulation that will be implemented from January 2025. JCA of oncological medicinal products and advanced therapies will be provided to Member States, but they will not include any ratings on ATV. Indeed, Member States remain responsible for drawing conclusions at the national level about the ATV of a health technology, as these conclusions depend on the specific healthcare context and the relevance of the individual analyses included in the JCA report (i.e., several comparators might be included in the JCA report, of which only a selection may be relevant to a particular Member State). In this framework, it is important that the assessment scope for JCA should be inclusive of all Member States’ needs in terms of data and analysis, and a clear and consensual definition of the key criteria for ATV evaluation and interpretation could be helpful in reducing access inequalities and harmonizing Member States’ assessments.

According to our primary analysis (all medicines appraised for innovativeness status in Italy), the highest level of agreement on ATV ratings was observed between the HAS and the G-BA, while the ATV assessments from AIFA appeared more generous. This result was found also when analyzing the most populated subgroups (oncological and orphan products). We performed a secondary analysis that focused on the appraisal of innovativeness by AIFA (i.e., an HTA assessment that includes also the therapeutic need and the quality of the presented evidence), and in this instance, the concordance rates between the three agencies rose sharply.

To our best knowledge, this is the first and most up-to-date study comparing ATV assessments among three European HTA agencies. A recent analysis applying different methodologies has compared AIFA and HAS and confirmed a low level of agreement between the two agencies on ATV assessments, while a higher agreement is reached for innovative medicinal products (14). Similarly, Rouf and colleagues (7) compared HAS and G-BA and found an overall homogeneity in ATV assessment ranking with some limited cases of divergent opinions through quali-quantitative analysis. On the contrary, Boucaud-Maitre and colleagues (15) compared decisions made by HAS and IQWiG (classification of ATV by four levels) and found a lower percentage of agreement (50 percent) compared to our analysis, although it should be noted that the latter study showed a divergence in ratings between the IQWiG’s early assessment and the G-BA’s final assessment on CAB.

The major strength of our analysis is represented by the performance of two separate analyses based on the Italian decisions. It has been shown that the therapeutic need does not correlate with the innovativeness decision taken by AIFA (11;12) and therefore the discrepancy between the two analyses is likely to be attributed to the quality of the evidence provided by applicants. Indeed, separating ATV from the quality of the evidence allows us to determine the potential of the drug in the first parameter while at the same time defining the “certainty” of this potential via the quality of the evidence realm. There is plenty of literature documenting a decrease in the quality of the evidence during pharmaceutical development, with an increase, among other elements, of single-arm studies and shorter trials, with outcomes that are not readily and directly quantitatively translatable into a clinical benefit (23). The quality of the evidence is embedded into the evaluation of the added value of medicines by the German and French HTA organizations, whereas the two assessment domains are separated in the Italian appraisal. When both are considered for Italy, that is when the analysis focused on medicines appraised as innovative or not innovative, the level of agreement increased, with a concordance rate between 72 percent and 82 percent.

Yet, the discrepancy between the two analyses also suggests that clinical trials are increasingly being developed to meet regulatory standards without providing sufficient evidence to reduce the uncertainties on the real added value of new drugs. Therefore, manufacturers should balance the risk of seeing a drug assessed less favorable by HTA organizations with the decreased risk of clinical failures, and health systems should balance more rapid access to medicines by patients with the uncertainty of the true benefit of these medicines.

The present work should be read in light of two main limitations. First, the dataset used only represents a subset of the new indications approved by the European Union (i.e., those that were perceived by the manufacturer to provide an important therapeutic benefit and that were therefore submitted for assessment of innovativeness to AIFA), which restricts the generalizability of the findings. It should, furthermore, be noted that innovativeness status is accessible only for severe disorders (a potentially fatal disorder, or a disorder that leads to repeated hospitalizations, or that causes disabilities that significantly compromise the quality of life of patients). The possibility that our results can be transferred to drugs with lower therapeutic potential or for less disabling disorders cannot be ascertained. Second, it should be acknowledged that the three assessments have different goals altogether which might explain part of the discrepancies. Innovativeness is appraised in Italy to get easier market access (dedicated resources and immediate access to regional markets). The ASMR and CAB are used to negotiate prices and price discount in France and Germany, respectively.

Notwithstanding our findings show that if ATV and the quality of the evidence are integrated, the level of agreement among HTA organizations is rather high.

This is a living study, and updates may be useful to monitor trends of alignment of European HTA agencies’ assessments over time. Greater transparency in AIFA’s evaluations of ATV for all medicines and not only for those that require innovativeness status, as is the case for HAS and G-BA, would allow for a broader sample size leading to greater generalizability of results. Finally, it would be interesting to include also other countries in this analysis, despite the existing variabilities.

## Conclusions

The comparison between AIFA, HAS, and G-BA assessments, which adopt a similar ATV classification, may be of value to understanding the different approaches and whether discrepancies in the assessment are systematic or by chance, thus informing European health policy-makers how to implement HTA harmonization rules on this topic.

The high degree of concordance (between 72 percent and 82 percent, considering the secondary analyses) between AIFA, HAS, and G-BA suggests that the system is now mature to make a JCA in most cases, avoiding duplication of assessments and reducing access inequalities.

## Supporting information

Casilli et al. supplementary material Casilli et al. supplementary material

## Supplementary material

The supplementary material for this article can be found at https://doi.org/10.1017/S026646232300048X.

Supplementary Table 1 – Therapeutic Added Value: AIFA, HAS, G-BA scores and definitions.

Supplementary Table 2 – a) Alternative classification in four levels and b) Supplementary analysis results.

Supplementary Table 3 – Active Principles and therapeutic indications evaluated by each agency.

Supplementary Figure 1 – Concordance on ATV assessments among HTA bodies - Subgroup analysis.
