# Supplementary material for: Do France, Germany, and Italy agree on the added therapeutic value of medicines?
Source: Int J Technol Assess Health Care. 2023 Aug 15;39(1):e54. doi: 10.1017/S026646232300048X (PMC11570191; doi:10.1017/S026646232300048X)
Supplement: Casilli et al. supplementary material [file S026646232300048Xsup001.pdf]

Supplementary Table 1. Therapeutic Added Value: AIFA, HAS, G-BA scores and definitions

**AIFA**		**HAS**		**G-BA**	
Score	*ATV*	*Score*	*ASMR*	*Score*	*CAB*
Maximum	*"Greater demonstrated efficacy on clinically relevant outcomes than therapeutic alternatives (when available). The drug is able to heal the disease or otherwise significantly modify its natural history"*	***Major***	*"For drugs with a new mechanism of action, which have demonstrated with a high level of evidence superiority associated with clinical efficacy in terms of mortality or morbidity over the clinically relevant comparator, in a context of insufficiently addressed medical need for a serious disease"*	***Major***	*"A significant and sustained improvement in therapeutic benefit that had not previously been achieved by the appropriate comparator, such as recovery from disease, a substantial a substantial increase in survival, long-term relief from severe symptoms, or major relief from serious side effects"*
Important	*"Greater demonstrated efficacy on clinically relevant outcomes, or ability to reduce the risk of disabling or potentially fatal complications, or better risk-to-benefit (R/B) ratio than alternatives, or avoidance of high-risk clinical procedures. The drug modifies the natural history of the disease in a subpopulation of patients, or otherwise represents a clinically relevant advantage, e.g., in terms of quality of life and disease-free interval compared with available therapeutic alternatives"*	***Considerable***	*"For drugs that have demonstrated superiority associated with clinical efficacy in terms of mortality or morbidity in a context of insufficiently addressed medical need. The value of this efficacy may be positively modulated by a substantial gain in quality of life and/or safety"*	***Important***	*"A considerable improvement in therapy relevant benefit not previously achieved by the appropriate comparator, in particular the attenuation of severe symptoms, a moderate survival gain, an improvement of the disease noticeable by patients, or a relevant relief from severe or other side effects"*
Moderate	*"Greater efficacy of moderate*	***Moderate***	*"For drugs that have*	***Minor***	*"A slight improvement in*

	magnitude or demonstrated in certain subpopulations of patients or on surrogate outcomes, and with limited effects on quality of life. For conditions in which the absence of a comparator is acceptable, availability of evidence suggesting better clinical efficacy and more favorable R/B profile than alternative available therapeutics"		*demonstrated superiority associated with clinical efficacy in terms of mortality or morbidity in a context of insufficiently addressed medical need. The value of this efficacy may be positively modulated by a substantial gain in quality of life and/or safety"*		*the therapy relevant benefit that was not previously achieved by relevant comparators, in particular a reduction in non-severe symptoms of the disease or a relevant relief from side effects"*
Poor	*"Greater efficacy that, however, has been demonstrated on outcomes that are not clinically relevant or is of low magnitude. Minor advantages (e.g., more favorable route of administration) compared with available therapeutic alternatives"*	***Minor***	*"For drugs that have demonstrated a small improvement in relation to the existing product. It reflects a non-optimal demonstration and/or quantity of effect (efficacy, quality of life, tolerance) given the medical context. It may be a drug that has demonstrated relevant efficacy with a slight and acceptable decrease in quality of life or tolerance. Alternatively, it may be a drug with little or non-optimal additional efficacy but with a gain in quality of life or tolerability."*	**Not proven**	*"Lack of proof of additional benefit"*
Absent	*"Absence of additional clinical benefit compared with therapeutic alternatives available"*	***Absent***	*"A demonstration based on a noninferiority study, a generic or biosimilar drug. In the*	**Less Benefit**	*"The benefit is less than those of the comparator"*

			absence of a therapeutic alternative or when alternatives are limited, it may also reflect a deficiency or uncertainty related to the choice of comparator, the quality of the evidence of effect, the quantity of the effect, or its clinical relevance"		
				Non-quantifiable	*"When the available scientific data does not allow its quantification"*

AIFA indicates Agenzia Italiana del Farmaco; ASMR, Amélioration du Service Médical Rendu; ATV, Added Therapeutic Value; CAB, Clinical Added Benefit; G-BA, Gemeinsame Bundesausschuss; HAS, Haute Autorité de Santé.

Supplementary Table 2. a) Alternative ATV classification in 4 levels and b) Alternative analysis results.

a)

	**AIFA**	**HAS**	**G-BA**
	ATV	***ASMR***	***CAB***
Major ATV	Maximum	Major/Considerable	Major
Important ATV	Important	Moderate	Important
Minor ATV	Moderate	Minor	Minor
No proof of benefit	Poor/Absent	Absent	Not Proven/Less Benfit

b)

	AIFA vs HAS	***AIFA vs G-BA***	***G-BA vs HAS***
Percentage agreement	46%	45%	50%
Cohen k-value	0,19	0,21	0,30
CI 95%	[0.08; 0.31]	[0.07; 0.35]	[0.16; 0.43]
N	159	101	101

AIFA indicates Agenzia Italiana del Farmaco; ASMR, Amélioration du Service Médical Rendu; ATV, Added Therapeutic Value; CAB, Clinical Added Benefit; G-BA, Gemeinsame Bundesausschuss; HAS, Haute Autorité de Santé.

Supplementary Table 3. Active Principles and therapeutic indications evaluated by each agency.

API	***THERAPEUTIC INDICATION***		
	**AIFA**	**HAS**	**G-BA**
crizanlizumab	*Is indicated for the prevention of recurrent vaso-occlusive crises (VOCs) in sickle cell disease patients aged 16 years and older. It can be given as an add-on therapy to hydroxyurea/hydroxycarbamide (HU/HC) or as monotherapy in patients for whom HU/HC is inappropriate or inadequate. The novel indication is limited to the treatment of patients with sickle cell disease aged 16 years and older who have presented with at least 2 VOC in the previous 12 months.*	*Is reimbursed for the prevention of recurrent vaso-occlusive crises (VOCs) in sickle cell disease patients aged 16 years and older. It can be given as an add-on therapy to hydroxyurea/hydroxycarbamide (HU/HC) or as monotherapy in patients for whom HU/HC is inappropriate or inadequate.*	*Is indicated for the prevention of recurrent vaso-occlusive crises (VOCs) in sickle cell disease patients aged 16 years and older. It can be given as an add-on therapy to hydroxyurea/hydroxycarbamide (HU/HC) or as monotherapy in patients for whom HU/HC is inappropriate or inadequate.*
brentuximab vedotin	*Is indicated for the treatment of adult patients with CD30+ cutaneous T-cell lymphoma (CTCL) after at least one prior systemic therapy.*	*Is indicated for the treatment of CD30+ mycosis fungoides (MF) and CD30+ cutaneous anaplastic large cell lymphoma (CALL) in adults after at least one prior systemic treatment.*	*Is indicated for the treatment of adult patients with CD30+ cutaneous T-cell lymphoma (CTCL) after at least one prior systemic therapy.*
brentuximab vedotin	*In combination with cyclophosphamide, doxorubicin and prednisone (CHP) is indicated for adult patients with previously untreated systemic anaplastic large cell lymphoma (sALCL).*	*Is indicated for the treatment of previously untreated systemic anaplastic large cell lymphoma (sALCL), in combination with cyclophosphamide, doxorubicin and prednisone, in the absence of an ALK mutation or in the presence of an ALK mutation (ALK+) in patients with an IPI score of ≥2.*	*In combination with cyclophosphamide, doxorubicin and prednisone (CHP) is indicated for adult patients with previously untreated systemic anaplastic large-cell lymphoma (sALCL).*
erenumab	*Is indicated for the prophylaxis of migraine in adults who have at least 4 migraine days per month.*	*Is indicated for the treatment of patients with severe migraine and at least 8 migraine days per month, with previous failure to at least two prophylactic treatments and without cardiovascular disease (patients having had a myocardial infarction, stroke, TIA, unstable angina or coronary artery bypass graft (CABG).*	*Is indicated for the prophylaxis of migraine in adults who have at least 4 migraine days per month.*
fremanezumab	*Is indicated for the prophylaxis of migraine in adults who have at least 4 migraine days per month.*	*Is indicated for the treatment of adult patients with severe migraine who have at least 8 migraine days per month, with previous failure to at least two prophylactic treatments*	*Is indicated for the prophylaxis of migraine in adults who have at least 4 migraine days per month.*

		and without cardiovascular disease (patients having had clinically significant cardiovascular disease or vascular ischaemia, or thromboembolic event).	
alectinib	*As monotherapy is indicated for the treatment of adult patients with ALK-positive advanced NSCLC previously treated with crizotinib.*	*As monotherapy is indicated for the treatment of advanced non-small cell lung cancer (NSCLC) with anaplastic lymphoma kinase (ALK-positive) gene rearrangement in adult patients previously treated with crizotinib.*	*As monotherapy is indicated for the treatment of adult patients with ALK-positive advanced NSCLC previously treated with crizotinib.*
alectinib	*As monotherapy is indicated for the first-line treatment of adult patients with anaplastic lymphoma kinase (ALK)-positive advanced non-small cell lung cancer (NSCLC).*	*As monotherapy is indicated for the first-line treatment of adults suffering from advanced metastatic non-small cell lung cancer (NSCLC) with ALK rearrangement.*	*As monotherapy is indicated for the first-line treatment of adult patients with anaplastic lymphoma kinase (ALK)-positive advanced non-small cell lung cancer (NSCLC).*
darvadstrocel	*Is indicated for the treatment of complex perianal fistulas in adult patients with non-active/mildly active luminal Crohn's disease when fistulas have shown an inadequate response to at least one conventional or biologic therapy. It should be used after conditioning of the fistulas.*	*Is indicated for the treatment of complex perianal fistulas in adult patients with non-active/mildly active luminal Crohn's disease when fistulas have shown an inadequate response to at least one conventional or biologic therapy.*	*Is indicated for the treatment of complex perianal fistulas in adult patients with non-active/mildly active luminal Crohn's disease when fistulas have shown an inadequate response to at least one conventional or biologic therapy. It should be used only after conditioning of the fistulas.*
brigatinib	*Is indicated as monotherapy for the treatment of adult patients with anaplastic lymphoma kinase (ALK)-positive advanced non-small cell lung cancer (NSCLC) previously not treated with an ALK inhibitor.*	*Is indicated as monotherapy for the treatment of adult patients with anaplastic lymphoma kinase (ALK)-positive advanced non-small cell lung cancer (NSCLC) previously not treated with a tyrosine kinase inhibitor that targets the ALK+ mutation.*	*Is indicated as monotherapy for the treatment of adult patients with anaplastic lymphoma kinase (ALK)-positive advanced non-small cell lung cancer (NSCLC) previously not treated with an ALK inhibitor.*
pegcetacoplan	*Is indicated in the treatment of adult patients with paroxysmal nocturnal hemoglobinuria (PNH) who remain anaemic after treatment with a C5 inhibitor for at least 3 months.*	*N.A.*	*Is indicated in the treatment of adult patients with paroxysmal nocturnal haemoglobinuria (PNH) who are anaemic after treatment with a C5 inhibitor for at least 3 months.*
glucagon	*Is indicated for the treatment of severe hypoglycemia in patients*	*Is indicated for the treatment of severe hypoglycaemia in adults, adolescents, and children aged 4*	*N.A.*

	with diabetes aged 4 years and older.	*years and over with type 1 diabetes mellitus or insulin-treated type 2 diabetes mellitus.*	
avelumab	*Is indicated as monotherapy for the treatment of adult patients with metastatic Merkel cell carcinoma (MCC).*	*Is indicated as monotherapy for the treatment of adult patients with metastatic Merkel cell carcinoma (MCC) previously treated with chemotherapy.*	*Is indicated as monotherapy for the treatment of adult patients with metastatic Merkel cell carcinoma (MCC).*
avelumab	*Is indicated as monotherapy for the first-line maintenance treatment of adult patients with locally advanced or metastatic urothelial carcinoma (UC) who are progression-free following platinum-based chemotherapy.*	*Is indicated as monotherapy for the first-line maintenance treatment of adult patients with locally advanced or metastatic urothelial carcinoma (UC) who are progression-free following platinum-based chemotherapy.*	*Is indicated as monotherapy for the first-line maintenance treatment of adult patients with locally advanced or metastatic urothelial carcinoma (UC) who are progression-free following platinum-based chemotherapy.*
inotuzumab ozogamicin	*Is indicated as monotherapy for the treatment of adults with relapsed or refractory CD22-positive B cell precursor acute lymphoblastic leukaemia (ALL). Adult patients with Philadelphia chromosome-positive (Ph+) relapsed or refractory B cell precursor ALL should have failed treatment with at least 1 tyrosine kinase inhibitor (TKI).*	*Is indicated for the treatment of adults with relapsed or refractory CD22 positive B-cell precursor acute lymphoblastic leukaemia (ALL), Adult patients with Philadelphia chromosome-positive (Phi+) should have failed treatment with at least 1 tyrosine kinase inhibitor (TKI).*	*Is indicated as monotherapy for the treatment of adults with relapsed or refractory CD22-positive B cell precursor acute lymphoblastic leukaemia (ALL). Adult patients with Philadelphia chromosome-positive (Ph+) relapsed or refractory B cell precursor ALL should have failed treatment with at least 1 tyrosine kinase inhibitor (TKI).*
belantamab mafodotin	*Is indicated as monotherapy for the treatment of multiple myeloma in adult patients, who have received at least four prior therapies and whose disease is refractory to at least one proteasome inhibitor, one immunomodulatory agent, and an anti-CD38 monoclonal antibody, and who have demonstrated disease progression on the last therapy.*	*Is indicated as monotherapy for the treatment of multiple myeloma in adult patients, who have received at least four prior therapies and whose disease is refractory to at least one proteasome inhibitor, one immunomodulatory agent, and an anti-CD38 monoclonal antibody, and who have demonstrated disease progression on the last therapy.*	*Is indicated as monotherapy for the treatment of multiple myeloma in adult patients, who have received at least four prior therapies and whose disease is refractory to at least one proteasome inhibitor, one immunomodulatory agent, and an anti-CD38 monoclonal antibody, and who have demonstrated disease progression on the last therapy.*
blinatumomab	*Is indicated for the treatment of paediatric patients aged 1 year and older with relapsed or refractory, CD19-positive, Philadelphia chromosome-negative B-cell precursor Acute Lymphoblastic Leukemia (ALL), relapsing after*	*Is indicated as monotherapy for the treatment of paediatric patients aged 1 year or older with Philadelphia chromosome-negative CD19 positive B-cell precursor ALL which is refractory or in relapse after receiving at least two prior*	*Is indicated as monotherapy for the treatment of paediatric patients aged 1 year or older with Philadelphia chromosome-negative, CD19-positive B-precursor ALL which is refractory or in relapse after receiving at least two prior*

	receiving at least two previous therapies or relapsing after hematopoietic stem cell allograft.	*therapies or in relapse after receiving prior allogeneic hematopoietic stem cell transplantation.*	*therapies or in relapse after receiving prior allogeneic hematopoietic stem cell transplantation.*
blinatumomab	*Is indicated as monotherapy for the treatment of paediatric patients aged 1 year and older with B-cell precursor LLA in first high-risk, CD19-positive, Philadelphia chromosome-negative relapse as part of consolidation therapy.*	*Is indicated as monotherapy for the treatment of children or adolescents aged 1 year or older with high-risk first relapsed Philadelphia chromosome-negative CD19 positive B-precursor acute lymphoblastic leukaemia (ALL) as part of the consolidation therapy.*	*Is indicated as monotherapy for the treatment of paediatric patients aged 1 year or older with high-risk first relapsed Philadelphia chromosome-negative CD19 positive B-precursor ALL as part of the consolidation therapy.*
blinatumomab	*Is indicated as monotherapy for the treatment of adults with Philadelphia chromosome-negative, CD19-positive (CD19+) B-cell precursor acute lymphoblastic leukaemia (BCP) (ALL) in first or second complete remission with minimal residual disease (MRD) greater than or equal to 0.1%.*	*N.A.*	*Is indicated as monotherapy for the treatment of adults with Philadelphia chromosome-negative, CD19-positive B-precursor ALL in first or second complete remission with minimal residual disease (MRD) greater than or equal to 0.1%.*
encorafenib	*Is indicated in combination with cetuximab for the treatment of adult patients with metastatic colorectal cancer (CRC) positive for the BRAF V600E mutation who have received previous systemic therapy.*	*Is indicated for the treatment of adult patients in combination with cetuximab with metastatic colorectal cancer with a BRAF V600E mutation, who have received prior systemic therapy.*	*Is indicated in combination with cetuximab, for the treatment of adult patients with metastatic colorectal cancer (CRC) with a BRAF V600E mutation, who have received prior systemic therapy.*
encorafenib	*Is indicated in combination with binimetinib is indicated for the treatment of adult patients with unresectable or metastatic melanoma with a BRAF V600 mutation.*	*Is indicated for the treatment of unresectable or metastatic melanoma in adults with a BRAF V600 mutation.*	*Is indicated in combination with binimetinib is indicated for the treatment of adult patients with unresectable or metastatic melanoma with a BRAF V600 mutation.*
cerliponase alfa	*Is indicated for the treatment of neuronal ceroid lipofuscinosis type 2 (CLN2) disease, also known as tripeptidyl peptidase 1 (TPP1) deficiency.*	*Is indicated for the treatment of neuronal ceroid lipofuscinosis type 2 (NCL2) or tripeptidyl peptidase-1 (TPP1) deficiency.*	*Is indicated for the treatment of neuronal ceroid lipofuscinosis type 2 (CLN2) disease, also known as tripeptidyl peptidase 1 (TPP1) deficiency.*
zanubrutinib	*As monotherapy is indicated for the treatment of adult patients with Waldenström's macroglobulinaemia (WM) who have received at least one prior therapy, or as first-line*	*N.A.*	*As monotherapy is indicated for the treatment of adult patients with Waldenström's macroglobulinaemia (WM) who have received at least one prior*

	treatment for patients ineligible for chemo-immunotherapy.		*therapy, or in first-line treatment for patients unsuitable for chemo-immunotherapy.*
odevixibat	*Is indicated for the treatment of progressive familial intrahepatic cholestasis (PFIC) in patients 6 months of age and older*	*Is indicated for the treatment of progressive familial intrahepatic cholestasis (PFIC) type 1 or 2 (with the exception of the BSEP3 subtype) in patients aged 6 months or older.*	*Is indicated for the treatment of progressive familial intrahepatic cholestasis (PFIC) in patients aged 6 months or older.*
caplacizumab	*Is indicated for the treatment of adults experiencing an episode of acquired thrombotic thrombocytopenic purpura (aTTP), in conjunction with plasma exchange and immunosuppression.*	*Is indicated for the treatment of adults experiencing an episode of acquired thrombotic thrombocytopenic purpura (aTTP), in conjunction with plasma exchange and immunosuppression.*	*Is indicated for the treatment of adults experiencing an episode of acquired thrombotic thrombocytopenic purpura (aTTP), in conjunction with plasma exchange and immunosuppression.*
cabozantinib	*Is indicated as monotherapy for the treatment of hepatocellular carcinoma (HCC) in adults who have previously been treated with sorafenib.*	*Is indicated as monotherapy in the treatment of adult patients with hepatocellular carcinoma (HCC) who have been treated with sorafenib.*	*Is indicated as monotherapy for the treatment of hepatocellular carcinoma (HCC) in adults who have previously been treated with sorafenib.*
burosumab	*Is indicated for the treatment of X-linked hypophosphatemia (XLH) with radiographic evidence of bone disease, in children one year of age and older and adolescents with a growing skeletal system.*	*Is indicated for the treatment of X-linked hypophosphatemia with radiographic evidence of bone involvement in children aged one year and older and in adolescents undergoing bone growth, with severe forms refractory to conventional therapy or with severe complicated forms.*	*Is indicated for the treatment of X-linked hypophosphatemia (XLH) with radiographic evidence of bone disease, in children one year of age and older and adolescents with a growing skeletal system.*
cisteamina cloridrato	*Is indicated for the treatment of cystine crystal deposits in the cornea in adults and children from 2 years of age with cystinosis.*	*Is indicated for the treatment of corneal cystine deposits in adults and children over 2 years of age with cystinosis.*	*N.A.*
daratumumab	*Is indicated in combination with lenalidomide and dexamethasone, or bortezomib and dexamethasone, for the treatment of adult patients with multiple myeloma who have received at least one prior therapy.*	*Is indicated in combination with lenalidomide and dexamethasone, or with bortezomib and dexamethasone, for the treatment of adults suffering from multiple myeloma and having received at least one prior treatment.*	*Is indicated in combination with lenalidomide and dexamethasone, or bortezomib and dexamethasone, for the treatment of adult patients with multiple myeloma who have received at least one prior therapy.*
daratumumab	*Is indicated as monotherapy for the treatment of adult patients with relapsed and refractory multiple*	*Is indicated as monotherapy for the treatment of adults suffering from relapsed and refractory multiple*	*Is indicated as monotherapy for the treatment of adult patients with relapsed and refractory multiple*

	myeloma, whose prior therapy included a proteasome inhibitor and an immunomodulatory agent and who have demonstrated disease progression on the last therapy.	*myeloma, for whom previous treatments included a proteasome inhibitor and an immunomodulator, and whose disease progressed during the last treatment.*	*myeloma, whose prior therapy included a proteasome inhibitor and an immunomodulatory agent and who have demonstrated disease progression on the last therapy.*
daratumumab	*Is indicated in combination with bortezomib, melphalan and prednisone for the treatment of adult patients with newly diagnosed multiple myeloma who are ineligible for autologous stem cell transplant.*	*Is indicated in combination with bortezomib, melphalan and prednisone for the treatment of adult patients with newly diagnosed multiple myeloma who are ineligible for autologous stem cell transplant*	*Is indicated in combination with bortezomib, melphalan and prednisone for the treatment of adult patients with newly diagnosed multiple myeloma who are ineligible for autologous stem cell transplant.*
daratumumab	*Is indicated in combination with lenalidomide and dexamethasone for the treatment of adult patients with newly diagnosed multiple myeloma who are ineligible for autologous stem cell transplant.*	*Is indicated in combination with lenalidomide and dexamethasone for the treatment of adult patients with newly diagnosed multiple myeloma who are ineligible for autologous stem cell transplant.*	*Is indicated in combination with lenalidomide and dexamethasone for the treatment of adult patients with newly diagnosed multiple myeloma who are ineligible for autologous stem cell transplant.*
doravirina/lamivudina /tenofovir disoproxil	*Is indicated for the treatment of adults infected with HIV 1 without past or present evidence of resistance to the NNRTI class, lamivudine, or tenofovir.*	*Is indicated for the treatment of HIV-1 infected adults with no previous or current evidence of resistance to the NNRTI class, lamivudine or tenofovir.*	*Is indicated for the treatment of adults infected with the human immunodeficiency virus (HIV-1). The HI viruses must not have mutations known to be associated with resistance to the NNRTI (non-nucleosidic reverse transcriptase inhibitor) class of substances, lamivudine, or tenofovir.*
avatrombopag	*Is indicated for the treatment of severe thrombocytopenia, in adult patients with chronic liver disease and scheduled to undergo an invasive procedure.*	*N.A.*	*Is indicated for the treatment of severe thrombocytopenia in adult patients with chronic liver disease who are scheduled to undergo an invasive procedure.*
dupilumab	*Is indicated for the treatment of adolescent patients aged 6 to 11 years with severe atopic dermatitis eligible for systemic therapy, who present with:*<br>*- EASI≥24*<br>*or any of the following characteristics:*<br>*1. Localization in visible and/or sensitive areas such as face/neck and/or hands and/or genitalia;*	*Is indicated for the treatment of severe atopic dermatitis in children 6 to 11 years old who are candidates for systemic therapy.*	*Is indicated for the treatment of severe atopic dermatitis in children 6 to 11 years old who are candidates for systemic therapy.*

	2. Assessment of itching with NRS≥7 scale;<br>*3. Quality of life assessment with CDLQI≥10 index*		
dupilumab	*Is indicated for the treatment of adolescent patients aged 12-17 years with severe atopic dermatitis eligible for systemic therapy who present with:*<br>*- EASI≥24*<br>*or any of the following characteristics:*<br>*1. Localization in visible and/or sensitive areas such as: face/neck and/or hands and/or genitalia;*<br>*2. Assessment of itching with NRS≥7 scale;*<br>*3.Assessment of quality of life with CDLQI≥10 index.3. Assessment of quality of life with CDLQI≥10 index.*	*Is indicated for the treatment of moderate to severe atopic dermatitis in adolescents 12 years and older who are candidates for systemic therapy.*	*Is indicated for the treatment of moderate-to-severe atopic dermatitis (AD) in adolescents 12 years and older who are candidates for systemic therapy.*
dupilumab	*Is indicated for the treatment of moderate-to-severe atopic dermatitis in adults who are candidates for systemic therapy. (This evaluation of innovativeness refers to the indication eligible for reimbursement: adult patients (age >18 years) with severe atopic dermatitis (AD) eligible for treatment with systemic therapy, for whom treatment with cyclosporine is contraindicated, ineffective, or not tolerated).*	*Is indicated for the treatment of moderate to severe atopic dermatitis (AD) in adults, requiring systemic treatment.*	*Is indicated for the treatment of moderate-to-severe atopic dermatitis (AD) in adolescents 12 years and older who are candidates for systemic therapy.*
galcanezumab	*Is indicated for the prophylaxis of migraine in adults who have at least 4 migraine days per month.*	*Is indicated for the treatment of patients with severe migraine who have at least 8 migraine days per month, with previous failure to at least two prophylactic treatments and without cardiovascular disease (patients having had a myocardial infarction, unstable angina, coronary artery bypass graft (CABG), percutaneous coronary intervention (PCI), stroke, deep-*	*Is indicated for the prophylaxis of migraine in adults who have at least 4 migraine days per month.*

		vein thrombosis (DVT) or other serious cardiovascular risk).	
satralizumab	*In adolescents 12 to <18 years of age: is indicated, either as monotherapy or in combination with immunosuppressive therapy (TIS) for the treatment of neuromyelitis optica spectrum disorders (NMOSD), in the presence of seropositivity for anti-aquaporin-4 IgG (AQP4-IgG) (as per licensed indication) and with baseline EDSS score ≤6.5. In patients starting treatment in adulthood: is indicated as a second-line treatment after rituximab, or in the case of contraindications to the use of rituximab, as monotherapy or in combination with immunosuppressive therapy for the treatment of neuromyelitis optica spectrum disorders (NMOSD) in the presence of seropositivity for anti-aquaporin-4 IgG (AQP4), clinical history of at least one relapse in the past 12 months, and an Expanded Disability Status Scale (EDSS) score ≤6.5.*	*Is indicated as a monotherapy or in combination with immunosuppressive therapy (IST) for the treatment of neuromyelitis optica spectrum disorders (NMOSD) only in adult and adolescent patients from 12 years of age who are anti-aquaporin-4 IgG (AQP4-IgG) seropositive, and who have a relapsing form of the disease not having responded to background immunosuppressive therapy (rituximab, azathioprine, mycophenolate mofetil).*	*Is indicated as a monotherapy or in combination with immunosuppressive therapy (IST) for the treatment of neuromyelitis optica spectrum disorders (NMOSD) in adult and adolescent patients from 12 years of age who are anti-aquaporin-4 IgG (AQP4-IgG) seropositive.*
cannabidiol	*Is indicated for use as adjunctive therapy of seizures associated with Lennox Gastaut syndrome (LGS), in conjunction with clobazam, for patients 2 years of age and older.*	*Is indicated as adjunctive therapy for seizures associated with Lennox-Gastaut syndrome (LGS) or Dravet syndrome (DS), in conjunction with clobazam, for patients 2 years of age and older.*	*Is is used in addition with clobazam, in patients two years of age and older for the adjuvant treatment of seizures associated with Lennox-Gastaut-Syndrome (LGS).*
cannabidiol	*Is indicated for use as adjunctive therapy of seizures associated with Dravet syndrome (DS), in conjunction with clobazam, for patients 2 years of age and older.*	*Is indicated as adjunctive therapy for seizures associated with Lennox-Gastaut syndrome (LGS) or Dravet syndrome (DS), in conjunction with clobazam, for patients 2 years of age and older.*	*Is used in addition with clobazam, in patients two years of age and older for the adjuvant treatment of seizures associated with Dravet syndrome (DS).*
risdiplam	*Is indicated for the treatment of spinal muscular atrophy (SMA) 5q in patients 2 months of age and older with a clinical diagnosis of*	*Is indicated for the treatment of 5q spinal muscular atrophy (SMA) in patients 2 months of age and older,*	*Is indicated for the treatment of 5q spinal muscular atrophy (SMA) in patients 2 months of age and older, with a clinical diagnosis of SMA*

	SMA type 1, type 2, or type 3 or having one to four copies of SMN2.	*with a clinical diagnosis of SMA type 1, type 2 or type 3.*	*Type 1, Type 2 or Type 3 or with one to four SMN2 copies.*
cefiderocol	*Is indicated for the treatment of infections due to aerobic Gram-negative organisms in adults with limited treatment options or with invasive infections with a strongly suspected aetiology of carbapenemase-resistant Gram-negative bacterial bacterium(according to the criteria identified in the prescribing chart).*	*Is indicated only as a last resort for the treatment of patients with infections due to multiresistant Gram-negative bacteria (particularly in the event of Enterobacterales and Pseudomonas aeruginosa, with a KPC, oxacillinase or Metallo-β-lactamase resistance mechanism [NDM, VIM, IMP]) and when the use of the other available options is not possible.*	*Is indicated for the treatment of infections due to aerobic Gram-negative organisms in adults with limited treatment options.*
fenfluramine	*Is indicated for the treatment of seizures associated with Dravet syndrome as adjunctive therapy to other antiepileptic drugs for patients 2 years of age and older.*	*Is indicated for the treatment of seizures associated with Dravet syndrome as an add-on therapy to other anti-epileptic medicines for patients 2 years of age and older.*	*Is indicated for the treatment of seizures associated with Dravet syndrome as an add-on therapy to other anti-epileptic medicines for patients 2 years of age and older.*
pralsetinib	*Is indicated as monotherapy for the treatment in lines after the first of adult patients with advanced non-small cell lung cancer (NSCLC) positive for the REarranged during Transfection (RET) gene fusion not previously treated with a RET inhibitor.*	*N.A.*	*Is indicated as monotherapy for the treatment in lines after the first of adult patients with advanced non-small cell lung cancer (NSCLC) positive for the REarranged during Transfection (RET) gene fusion not previously treated with a RET inhibitor.*
obinutuzumab	*In combination with chemotherapy, followed by obinutuzumab maintenance therapy in patients achieving a response, is indicated for the treatment of patients with previously untreated advanced follicular lymphoma.*	*In combination with induction chemotherapy followed by obinutuzumab maintenance in responders is indicated for patients with previously untreated advanced follicular lymphoma.*	*In combination with chemotherapy, followed by obinutuzumab maintenance therapy in patients achieving a response, is indicated for the treatment of patients with previously untreated advanced FL.*
fingolimod	*Is indicated as a single disease-modifying therapy in highly active relapsing-remitting multiple sclerosis for the following groups of adult patients and paediatric patients aged 10 years and older: - Patients with highly active disease despite a full and adequate course of treatment with at least one disease-modifying therapy*	*Is indicated as monotherapy for the background treatment of very active relapsing-remitting multiple sclerosis (RRMS) in the following groups of paediatric patients aged 10 years and older: - patients with very active disease despite complete and well-conducted treatment with at least one disease-modifying therapy for MS, or -patients with*	*Is indicated in children and adolescents ≥ 10 and < 18 years of age with highly active relapsing-remitting multiple sclerosis.*

	or<br>*- Patients with rapidly evolving severe relapsing-remitting multiple sclerosis defined by 2 or more disabling relapses in one year, and with 1 or more Gadolinium enhancing lesions on brain MRI or a significant increase in T2 lesion load as compared to a previous recent MRI.*	*severe and rapidly progressing RRMS, defined as 2 or more disabling relapses in one year associated with 1 or more enhanced lesion(s) after Gadolinium injection on brain MRI or a significant increase in T2 lesion load compared to a recent previous MRI.*	
givosiran	*Is indicated for the treatment of acute hepatic porphyria (AHP) in adults and adolescents aged 12 years and older.*	*Is indicated for the treatment of patients aged 18 years and over with acute hepatic porphyria (AHP) and with active disease (at least 2 porphyria attacks requiring hospitalization, an urgent healthcare visit or treatment with IV hemin at home, in the past 6 months).*	*Is indicated for the treatment of acute hepatic porphyria (AHP) in adults and adolescents aged 12 years and older.*
emicizumab	*Is indicated for routine prophylaxis of bleeding episodes in patients with severe haemophilia A (congenital factor VIII deficiency, FVIII < 1%) without factor VIII inhibitors. It can be used in all age groups.*	*Is indicated for prophylaxis to prevent bleeding episodes in patients with severe haemophilia A (congenital factor VIII deficiency, FVIII < 1%) without an anti-factor VIII inhibitor. It can be used in all age groups.*	*Is indicated for routine prophylaxis of bleeding episodes in patients with severe haemophilia A (congenital factor VIII deficiency, FVIII < 1%) without factor VIII inhibitors. It can be used in all age groups.*
emicizumab	*Is indicated for routine prophylaxis of bleeding episodes in patients with haemophilia A with factor VIII inhibitors. It can be used in all age groups*	*Is indicated for the prevention of bleeding episodes in patients with haemophilia A who have developed an anti-factor VIII inhibitor.*	*Is indicated for routine prophylaxis of bleeding episodes in patients with haemophilia A with factor VIII inhibitors. It can be used in all age groups*
adalimumab	*Is indicated for the treatment of noninfectious intermediate, posterior, and panuveitis uveitis in adult patients who have had an inadequate response to corticosteroids, in patients who require corticosteroid-sparing drugs, or in whom corticosteroid treatment is inappropriate.*	*Is indicated for the treatment of non-infectious intermediate, posterior and panuveitis in adults who have had an inadequate response to corticosteroids, in patients in need of corticosteroid-sparing, or in whom corticosteroid treatment is inappropriate.*	*N.A.*
palbociclib	*Is indicated for the treatment of hormone receptor (HR) positive, human epidermal growth factor*	*Is indicated for the treatment of locally advanced or metastatic hormone receptor (HR)-positive*	*Is indicated for the treatment of hormone receptor (HR) positive, human epidermal growth factor*

	receptor 2 (HER2) negative locally advanced or metastatic breast cancer in combination with an aromatase inhibitor.	*and human epidermal growth factor receptor-2 (HER2-)-negative breast cancer in combination with an aromatase inhibitor.*	*receptor 2 (HER2) negative locally advanced or metastatic breast cancer in combination with an aromatase inhibitor. In pre- or perimenopausal women, endocrine therapy should be combined with a luteinizing hormone-releasing hormone (LHRH) agonist.*
palbociclib	*Is indicated for the treatment of hormone receptor (HR) positive, human epidermal growth factor receptor 2 (HER2) negative locally advanced or metastatic breast cancer in combination with fulvestrant in women who have received prior endocrine therapy.*	*Is indicated for the treatment of locally advanced or metastatic, HR+ and HER2-negative breast cancer: in combination with an aromatase inhibitor; in combination with fulvestrant in women previously treated with hormonal therapy.*	*Is indicated for the treatment of hormone receptor (HR) positive, human epidermal growth factor receptor 2 (HER2) negative locally advanced or metastatic breast cancer in combination with fulvestrant in women who have received prior endocrine therapy. In pre- or perimenopausal women, endocrine therapy should be combined with a luteinizing hormone-releasing hormone (LHRH) agonist.*
imlifidase	*Is indicated for the desensitization treatment of highly sensitized adult patients in need of kidney transplantation with a positive crossmatch against an available deceased donor. Its use should be reserved for patients who are unlikely to undergo transplantation within the available kidney allocation system, including priority allocation programs for highly sensitized patients.*	*Is indicated for the desensitisation treatment of highly sensitised adult kidney transplant patients with positive crossmatch against an available deceased donor, with use to be reserved for patients unlikely to be transplanted under the current available kidney allocation system, including prioritisation programmes for highly sensitised patients.*	*Is indicated for the desensitisation treatment of highly sensitised adult kidney transplant patients with positive crossmatch against an available deceased donor. Its use should be reserved for patients unlikely to be transplanted under the available kidney allocation system including prioritisation programmes for highly sensitised patients.*
canakinumab	*Is indicated for the treatment of the following autoinflammatory periodic fever syndromes in adults, adolescents and children of 2 years of age and older: Tumor necrosis factor receptor (TNF)-associated periodic syndrome (TRAPS); Hyperimmunoglobulinemia D syndrome (HIDS)/mevalonate kinase deficiency (MKD); Familial Mediterranean fever (FMF). It should be administered in*	*Is indicated for the treatment of three forms of hereditary recurrent fevers in adults, adolescents and children aged 2 years and older: TNF-associated periodic syndrome (TRAPS), hyperimmunoglobulin D syndrome (HIDS)/mevalonate kinase deficiency (MKD), and familial Mediterranean fever (FMF).*	*N.A.*

	combination with colchicine, if appropriate. Relative to the indication of Familial Mediterranean Fever (FMF), reimbursability is limited to the "treatment of patients who do not respond to or are intolerant to colchicine".		
ibrutinib	*As a single agent is indicated for the treatment of adult patients with previously untreated chronic lymphocytic leukaemia.*	*As a single agent is indicated for the treatment of adult patients with previously untreated chronic lymphocytic leukaemia (CLL), only in patients not eligible for treatment with full-dose fludarabine.*	*As a single agent is indicated for the treatment of adult patients with previously untreated chronic lymphocytic leukaemia (CLL).*
ibrutinib	*In combination with rituximab is indicated for the treatment of adult patients with previously untreated chronic lymphocytic leukaemia.*	*In combination with rituximab for the first-line treatment of chronic lymphocytic leukaemia (CLL), only in patients eligible for full-dose fludarabine treatment and not presenting a del17p deletion or a TP53 mutation.*	*In combination with rituximab is indicated for the treatment of adult patients with previously untreated chronic lymphocytic leukaemia (CLL).*
ibrutinib	*In combination with bendamustine and rituximab (BR) is indicated for the treatment of adult patients with CLL who have received at least one previous therapy.*	*N.A.*	*In combination with bendamustine and rituximab (BR) is indicated for the treatment of adult patients with CLL who have received at least one previous therapy.*
setmelanotide	*Is indicated for the treatment of obesity and hunger control associated with pro-opiomelanocortin (POMC) deficiency, including PCSK1, with genetically confirmed bi-allelic loss of function, or bi-allelic leptin receptor (LEPR) deficiency in adults and children aged 6 years and older.*	*Is indicated for the treatment of obesity and control of hunger associated with genetically confirmed loss of biallelic pro-opiomelanocortin (POMC) function, including PCSK1 deficiency or biallelic leptin receptor deficiency (LEPR), in adults and children aged 6 years and older.*	*Is indicated for the treatment of obesity and hunger control associated with pro-opiomelanocortin (POMC) deficiency, including PCSK1, with genetically confirmed bi-allelic loss of function, or bi-allelic leptin receptor (LEPR) deficiency in adults and children aged 6 years and older.*
durvalumab	*As monotherapy is indicated for the treatment of locally advanced, unresectable non-small cell lung cancer (NSCLC) in adults whose tumours express PD-L1 on ≥ 1% of tumour cells and whose disease has*	*Is indicated for the treatment of locally advanced, non-operable non-small cell lung cancer (NSCLC) that has not progressed at the end of concurrent chemoradiotherapy in patients in*	*As monotherapy is indicated for the treatment of locally advanced, unresectable non-small cell lung cancer (NSCLC) in adults whose tumours express PD-L1 on ≥ 1% of tumour cells and whose disease has not progressed following*

	not progressed following platinum-based chemoradiation therapy.	*good general condition and whose tumours express PD-L1 ≥ 1%.*	*platinum-based chemoradiation therapy.*
dostarlimab	*Is indicated as monotherapy for the treatment of adult patients with advanced or recurrent endometrial carcinoma with Mismatch Repair (dMMR)/elevated microsatellite instability (MSI-H) system deficiency that progressed during or after previous treatment with a platinum-based regimen.*	*N.A.*	*Is indicated as monotherapy for the treatment of adult patients with mismatch repair deficient (dMMR)/microsatellite instability-high (MSI-H) recurrent or advanced endometrial cancer (EC) that has progressed on or following prior treatment with a platinum-containing regimen.*
trastuzumab emtansine	*As monotherapy, is indicated for the adjuvant treatment of adult patients with early-stage HER2-positive breast cancer with residual invasive disease in the breast and/or lymph nodes after neoadjuvant taxane and anti-HER2-targeted therapy.*	*Is indicated for the adjuvant treatment of adult patients with HER2-positive early breast cancer who have residual invasive disease, in the breast and/or lymph nodes, after neoadjuvant taxane-based and HER2-targeted therapy.*	*As a single agent, is indicated for the adjuvant treatment of adult patients with HER2-positive early breast cancer who have residual invasive disease, in the breast and/or lymph nodes, after neoadjuvant taxane-based and HER2-targeted therapy.*
ivacaftor/tezacaftor/elexacaftor	*Is indicated in a combination regimen with ivacaftor 150 mg tablets, for the treatment of cystic fibrosis (CF) in patients aged 12 years and older, who are heterozygous for F508del in the CFTR gene with a mutation: either gating (F/G genotype) or residual function (F/RF genotype) or unclassified (F/unclassified genotype) or unidentified (F/unidentified genotype).*	*Is indicated for the treatment of cystic fibrosis (CF) in patients aged 12 years and older who are heterozygous for the F508del mutation in the cystic fibrosis transmembrane conductance regulator (CFTR) gene and who have one of the gating mutations or a residual function mutation.*	*Is indicated in a combination regimen with ivacaftor 150 mg tablets for the treatment of cystic fibrosis in subjects aged 12 years and older who are heterozygous for the F508del mutation in the CFTR gene and carry a mutation on the second allele, which is not a minimal function, no gating (including R117H) and no residual function mutation, or the mutation on the second allele is unknown (other mutations).*
ivacaftor/tezacaftor/elexacaftor	*Is indicated in a combination regimen with ivacaftor for the treatment of cystic fibrosis (CF) in patients aged 6 to < 12 years who are homozygous for the F508 mutation in the gene for the cystic fibrosis transmembrane conductance regulator (CFTR) (F/F genotype) or heterozygous with a minimal function mutation (F/MF genotype).*	*Is indicated for the treatment of cystic fibrosis patients aged 6 to 11 years homozygous for the F508del mutation of the CFTR gene or heterozygous for the F508del mutation of the CFTR gene and carrying a minimal function CFTR gene mutation.*	*Is indicated in a combination regimen with ivacaftor for the treatment of cystic fibrosis in patients aged 6 to 11 years who are homozygous for an F508del mutation in the CFTR gene.*

ivacaftor/tezacaftor/elexacaftor	*Is indicated in a combination regimen with ivacaftor for the treatment of cystic fibrosis (CF) in patients aged 6 to < 12 years who are heterozygous for the F508del mutation in the gene for the cystic fibrosis transmembrane conductance regulator (CFTR) (genotypes F/RF, F/G, F/un classified, F/unidentified).*	*Is indicated for the treatment of cystic fibrosis patients aged 6 to 11 years heterozygous for the F508del mutation of the CFTR gene and carrying a residual function mutation or a so-called "gating" mutation.*	*Is indicated in a combination regimen with ivacaftor for the treatment of cystic fibrosis in patients aged 6 to 11 years who are heterozygous for an F508del mutation in the CFTR gene and carry a residual function mutation on the second allele.*
ivacaftor/tezacaftor/elexacaftor	*Is indicated in a combination regimen with ivacaftor 150 mg tablets for the treatment of cystic fibrosis (CF) in patients aged 12 years and older who are homozygous for the F508 mutation in the gene for the cystic fibrosis transmembrane conductance regulator (CFTR).*	*Is indicated in combination with ivacaftor for the treatment of cystic fibrosis (CF) in patients aged 12 years and older who are homozygous for the F508del mutation in the cystic fibrosis transmembrane conductance regulator (CFTR) gene or heterozygous for F508del in the CFTR gene with a minimal function (MF) mutation.*	*N.A.*
ivacaftor/tezacaftor/elexacaftor	*Is indicated in a combination regimen with ivacaftor 150 mg tablets for the treatment of cystic fibrosis (CF) in patients aged 12 years and older who are heterozygous for F508 in the gene for the cystic fibrosis transmembrane conductance regulator (CFTR) with a minimal function (MF) mutation.*	*Is indicated in combination with ivacaftor for the treatment of cystic fibrosis (CF) in patients aged 12 years and older who are homozygous for the F508del mutation in the cystic fibrosis transmembrane conductance regulator (CFTR) gene or heterozygous for F508del in the CFTR gene with a minimal function (MF) mutation.*	*Is indicated in a combination regimen with ivacaftor 150 mg tablets for the treatment of cystic fibrosis (CF) in subjects aged 12 years and older, who are heterozygous for the F508del mutation in the CFTR gene and carry a residual function mutation on the second allele.*
ivacaftor	*Is indicated in a combination regimen with ivacaftor/tezacaftor/elexacaftor tablets, for the treatment of cystic fibrosis (CF) in patients aged 12 years and older, who are heterozygous for F508 in the CFTR gene with a mutation: either gating (F/G genotype) or residual function (F/RF genotype) or unclassified (F/unclassified genotype) or*	*Is indicated for the treatment of cystic fibrosis patients aged 12 years and older, heterozygous for the F508del mutation of the CFTR gene and carrying one of the gating or residual function mutations.*	*Tablets are used as part of a combination regimen with ivacaftor/ tezacaftor/ elexacaftor tablets for the treatment of cystic fibrosis in subjects aged 12 years and older, who are heterozygous for the F508del mutation in the CFTR gene and carry a residual function mutation on the second allele.*

	unidentified (F/unidentified genotype).		
ivacaftor	*Tablets are used in a combination regimen with ivacaftor/tezacaftor/elexacaftor tablets for the treatment of adults, adolescents, and children aged six and older and younger than 12 with cystic fibrosis (CF) who have at least one F508 mutation in the CFTR gene (F/F genotype) or heterozygotes with a minimal function mutation (F/MF genotype).*	*Is indicated for the treatment of cystic fibrosis patients aged 6 to 11 years homozygous for the F508del mutation of the CFTR gene or heterozygous for the F508del mutation of the CFTR gene and carrying a minimal function mutation of the CFTR gene.*	*Is indicated in a combination regimen with ivacaftor/ tezacaftor/ elexacaftor for the treatment of children aged 6 to 11 years with cystic fibrosis, who are heterozygous for an F508del mutation in the CFTR gene and carry a minimal function mutation on the second allele.*
ivacaftor	*Is indicated in a combination regimen with ivacaftor/tezacaftor/elexacaftor for the treatment of cystic fibrosis (CF) in patients aged 6 to < 12 years who are heterozygous for the F508 mutation in the gene for the cystic fibrosis transmembrane conductance regulator (CFTR) (genotypes F/RF, F/G, F/unclassified, F/unidentified).*	*Is indicated for the treatment of cystic fibrosis patients aged 6 to 11 years who are heterozygous for the F508del mutation in the CFTR gene and carry a residual function mutation or a gating mutation.*	*Tablets are indicated in a combination regimen with ivacaftor/ tezacaftor/ elexacaftor tablets for the treatment of cystic fibrosis in patients aged 6 to 11 years who are heterozygous for the F508del mutation in the CFTR gene and carry a gating mutation (including R117H) on the second allele.*
ivacaftor	*Is indicated in a combination regimen with ivacaftor 75 mg/tezacaftor 50 mg/elexacaftor 100 mg tablets for the treatment of adults and adolescents aged 12 years and older with cystic fibrosis (CF) homozygotes who have at least one F508 mutation in the CFTR gene.*	*In combination with ivacaftor for the treatment of cystic fibrosis (CF) in patients aged 12 years and older who are homozygous for the F508del mutation in the cystic fibrosis transmembrane conductance regulator (CFTR) gene or heterozygous for F508del in the CFTR gene with a minimal function (MF) mutation.*	*Is indicated in patients 12 years of age and older with cystic fibrosis who are heterozygous for the F508del mutation and who display one of the following mutations in the CFTR gene: P67L, R117C, L206W, R352Q, A455E, D579G, 711+3A→G, S945L, S977F, R1070W, D1152H, 2789+5G→A, 3272-26A→G and 3849+10kbC→T.*
ivacaftor	*Is indicated in in a combination regimen with ivacaftor/tezacaftor/elexacaftor tablets for the treatment of adults and adolescents aged 12 years and older with cystic fibrosis (CF) heterozygotes who have at least one F508 mutation in the CFTR gene*	*In combination with ivacaftor for the treatment of cystic fibrosis (CF) in patients aged 12 years and older who are homozygous for the F508del mutation in the cystic fibrosis transmembrane conductance regulator (CFTR) gene or heterozygous for F508del in the CFTR gene with a minimal function (MF) mutation.*	*Tablets are used in the framework of a combination regimen with ivacaftor/tezacaftor/elexacaftor tablets for the treatment of adults and adolescents aged 12 years and older with cystic fibrosis (CF) who are heterozygous for F508del and*

	with a minimal function (MF) mutation.		*have a minimal function (MF) mutation in the CFTR gene.*
pembrolizumab	*As monotherapy or in combination with platinum and 5 fluorouracil (5 FU) chemotherapy, is indicated for the first-line treatment of metastatic or unresectable recurrent head and neck squamous cell carcinoma in adults whose tumours express PD L1 with a CPS ≥ 1.*	*Is indicated as monotherapy or in combination with platinum and 5-fluorouracil (5-FU) chemotherapy in the first-line treatment of metastatic or unresectable recurrent head and neck squamous cell carcinoma (HNSCC) in adults whose tumours express PD-L1 with a CPS ≥ 1.*	*As monotherapy or in combination with platinum and 5-fluorouracil (5-FU) chemotherapy, is indicated for the first-line treatment of metastatic or unresectable recurrent head and neck squamous cell carcinoma in adults whose tumours express PD-L1 with a CPS ≥ 1.*
pembrolizumab	*In combination with carboplatin and either paclitaxel or nab-paclitaxel, is indicated for the first-line treatment of metastatic squamous non-small cell lung carcinoma in adults. The reimbursability of the indication is limited to patients with PD-L1 expression level<50%.*	*Is indicated in combination with carboplatin and either paclitaxel or nab-paclitaxel as a first-line treatment for adult patients with metastatic squamous non-small cell lung carcinoma (NSCLC).*	*In combination with carboplatin and either paclitaxel or nab-paclitaxel, is indicated for the first-line treatment of metastatic squamous NSCLC in adults whose tumours express PD-L1 with a < 50% tumour proportion score (TPS).*
pembrolizumab	*As monotherapy is indicated for the treatment of adult patients with relapsed or refractory classical Hodgkin's lymphoma (cHL) who have failed treatment with autologous stem cell transplantation (ASCT) and brentuximab vedotin (BV), or who are ineligible for transplantation and have failed treatment with BV.*	*Is indicated for the treatment of adults with relapsed or refractory classical Hodgkin's lymphoma (HLc) in 2 situations: after failure of autologous stem cell transplantation (ASCT) and brentuximab vedotin (BV) failure, or in patients ineligible for transplantation and after BV treatment failure.*	*As monotherapy is indicated for the treatment of adult patients with relapsed or refractory classical Hodgkin's lymphoma (cHL) who have failed treatment with autologous stem cell transplantation (ASCT) and brentuximab vedotin (BV), or who are ineligible for transplantation and have failed treatment with BV.*
pembrolizumab	*As monotherapy is indicated for the treatment of locally advanced or metastatic urothelial carcinoma in adults who have received prior platinum-containing chemotherapy.*	*Is indicated as monotherapy for the treatment of adults with locally advanced or metastatic urothelial cancer having received prior platinum salt-based chemotherapy.*	*Is indicated as monotherapy for the treatment of locally advanced or metastatic urothelial carcinoma after prior platinum-based therapy in adults.*
pembrolizumab	*In combination with pemetrexed and platinum chemotherapy, is indicated for the first-line treatment of metastatic non-squamous non-small cell lung carcinoma in adults whose tumours have no EGFR or ALK positive mutations and having an expression level of PD-L1<50%.*	*Is indicated in combination with pemetrexed and platinum salt chemotherapy, in the first-line treatment of adult patients with metastatic non-squamous NSCLC whose tumours do not have EGFR or ALK mutations.*	*In combination with pemetrexed and platinum chemotherapy, is indicated for the first-line treatment of metastatic non-squamous non-small cell lung carcinoma in adults whose tumours have no EGFR or ALK positive mutations and having an expression level of PD-L1<50%.*

pembrolizumab	*As monotherapy is indicated in the adjuvant treatment of adult patients with Stage III melanoma and lymph node involvement who have undergone complete resection.*	*Is indicated as adjuvant treatment of adult patients with node-positive stage III melanoma who have undergone complete resection.*	*As monotherapy is indicated for the adjuvant treatment of adults with Stage III melanoma and lymph node involvement who have undergone complete resection.*
pembrolizumab	*In combination with axitinib, is indicated for the first-line treatment of advanced renal cell carcinoma in adults*	*Is indicated in combination with axitinib in the first-line treatment of advanced purely clear-cell renal cell carcinoma (RCC) or with a clear-cell component.*	*In combination with axitinib, is indicated for the first-line treatment of advanced renal cell carcinoma in adults*
pembrolizumab	*As monotherapy is indicated in the first-line treatment of metastatic colorectal cancer with microsatellite instability-high (MSI-H) or mismatch repair deficient (dMMR) in adults.*	*Is indicated as monotherapy for the first-line treatment of metastatic microsatellite instability-high (MSI-H) or mismatch repair deficient (dMMR) colorectal cancer in adults, only in patients unresectable from the outset.*	*As monotherapy is indicated for the first-line treatment of metastatic microsatellite instability-high (MSI-H) or mismatch repair deficient (dMMR) colorectal cancer in adults.*
pembrolizumab	*As monotherapy is indicated for the treatment of adult and pediatric patients aged 3 years and older with relapsed or refractory classical Hodgkin's lymphoma (r/r cHL) who have failed treatment with autologous stem cell transplantation (ASCT) or following at least two prior therapies when ASCT is not a treatment option.*	*Is indicated as monotherapy in the treatment of adult and paediatric patients aged 3 years and older with relapsed or refractory classical Hodgkin's lymphoma after failure of autologous stem cell transplantation (ASCT) or after at least two prior lines of treatment when autologous transplantation is not a treatment option.*	*As monotherapy is indicated for the treatment of adult and paediatric patients aged 3 years and older with relapsed or refractory classical Hodgkin lymphoma who have failed autologous stem cell transplant (ASCT) or following at least two prior therapies when ASCT is not a treatment option.*
ribociclib	*In combination with an aromatase inhibitor is indicated as initial endocrine-based therapy for the treatment of postmenopausal women with locally advanced or metastatic hormone receptor (HR)-positive and human epidermal growth factor receptor 2 (HER2)-negative breast cancer.*	*Is indicated in combination with fulvestrant in the treatment of postmenopausal women with hormone receptor (HR)-positive, human epidermal growth factor receptor 2 (HER2)-negative locally advanced or metastatic breast cancer, in the absence of short-term life-threatening symptomatic visceral involvement, as initial endocrine-based therapy, or in women who have received prior endocrine therapy.*	*Is indicated in post-menopausal women with hormone receptor (HR)-positive, HER2-negative locally advanced or metastatic breast cancer who have not yet received initial endocrine therapy.*
ribociclib	*Is indicated for the treatment of women with hormone receptor (HR)-positive, human epidermal*	*In combination with fulvestrant is indicated in the treatment of postmenopausal women with*	*Is indicated for the treatment of women with hormone receptor (HR)-positive, human epidermal*

	growth factor receptor 2 (HER2)-negative locally advanced or metastatic breast cancer in combination with an aromatase inhibitor or fulvestrant as initial endocrine-based therapy, or in women who have received prior endocrine therapy. In pre- or perimenopausal women, the endocrine therapy should be combined with a luteinising hormone-releasing hormone (LHRH) agonist. (Therapeutic innovation about the following indications: - in combination with fulvestrant in postmenopausal patients; - in combination with aromatase inhibitors in pre-perimenopausal patients.	*hormone receptor (HR)-positive, human epidermal growth factor receptor 2 (HER2)-negative locally advanced or metastatic breast cancer, in the absence of short-term life-threatening symptomatic visceral involvement, as initial endocrine-based therapy, or in women who have received prior endocrine therapy.*	*growth factor receptor 2 (HER2)-negative locally advanced or metastatic breast cancer in combination with an aromatase inhibitor or fulvestrant as initial endocrine-based therapy, or in women who have received prior endocrine therapy. In pre- or perimenopausal women, the endocrine therapy should be combined with a luteinising hormone-releasing hormone (LHRH) agonist.*
tisagenlecleucel	*Is indicated for the treatment of Paediatric and young adult patients up to and including 25 years of age with B cell acute lymphoblastic leukaemia (ALL) that is refractory, in relapse post-transplant or in second or later relapse.*	*Is indicated for the treatment of children and young adults up to the age of 25 years suffering from refractory B-cell ALL, relapsed after transplantation, or after the second or later relapse.*	*Is indicated for the treatment of: Paediatric and young adult patients up to and including 25 years of age with B-cell acute lymphoblastic leukaemia (ALL) that is refractory, in relapse post-transplant or in second or later relapse.*
tisagenlecleucel	*Is indicated for the treatment of adult patients with relapsed or refractory diffuse large B cell lymphoma (DLBCL) after two or more lines of systemic therapy.*	*Is indicated for the treatment of adult patients with relapsed or refractory diffuse large B-cell lymphoma after two or more lines of systemic therapy.*	*Is indicated for the treatment of adult patients with relapsed or refractory diffuse large B-cell lymphoma (DLBCL) after two or more lines of systemic therapy.*
carfilzomib	*In combination with daratumumab and dexamethasone is indicated for the treatment of adult patients with multiple myeloma who have already undergone at least one previous therapy.*	*In combination with daratumumab and dexamethasone is indicated for the treatment of adult patients with multiple myeloma who have received at least one prior treatment.*	*In combination with daratumumab and dexamethasone, with lenalidomide and dexamethasone, or with dexamethasone alone is indicated for the treatment of adult patients with multiple myeloma who have received at least one prior therapy.*
velmanase alfa	*Is indicated for enzyme replacement therapy for the treatment of non-neurological manifestations in*	*Is indicated for the enzyme replacement therapy of non-neurological manifestations in*	*Is indicated for enzyme replacement therapy for the treatment of non-neurological manifestations in*

	patients with mild to moderate alpha-mannosidosis.	*patients suffering from mild to moderate alpha-mannosidosis.*	*patients with mild to moderate alpha-mannosidosis.*
lenvatinib	*Is indicated as monotherapy for the treatment of adult patients with advanced or unresectable hepatocellular carcinoma (HCC) who have received no prior systemic therapy.*	*N.A.*	*Is indicated as monotherapy for the treatment of adult patients with advanced or unresectable hepatocellular carcinoma (HCC) who have received no prior systemic therapy.*
atidarsagene autotemcel	*Is indicated for the treatment of metachromatic leukodystrophy (MLD) characterized by biallelic mutations in the arylsulfatase A (ARSA) gene that result in reduced ARSA enzyme activity:*<br>*-in children with late infantile or early juvenile forms without clinical manifestations of the disease,*<br>*-in children with the early juvenile form, with early clinical manifestations of the disease, who retain the ability to walk independently and before the onset of cognitive decline.*	*N.A.*	*Is indicated for the treatment of metachromatic leukodystrophy (MLD), characterised by biallelic mutations in the arylsulfatase A (ARSA) gene, leading to a reduction of the ARSA enzymatic activity: in children with late infantile or early juvenile forms, without clinical manifestations of the disease; in children with the early juvenile form, with early clinical manifestations of the disease, who still have the ability to walk independently and before the onset of cognitive decline.*
cemiplimab	*As monotherapy is indicated for the treatment of adult patients with metastatic or locally advanced cutaneous squamous cell carcinoma who are not candidates for curative surgery or curative radiotherapy.*	*Is indicated for the treatment of adult patients with metastatic or locally advanced cutaneous squamous cell carcinoma who are not candidates for curative surgery or curative radiation.*	*As monotherapy is indicated for the treatment of adult patients with locally advanced or metastatic basal cell carcinoma (laBCC or mBCC) who have progressed on or are intolerant to a hedgehog pathway inhibitor (HHI).*
lutetium-(177Lu)-oxodotreotide	*Is indicated in adult patients for the treatment of well-differentiated (G1 and G2), progressive, non excisable or metastatic, somatostatin receptor-positive gastroenteropancreatic neuroendocrine tumours (NET-GEP).*	*Is indicated for the treatment of well-differentiated (G1 and G2), progressive, unresectable or metastatic gastroenteropancreatic neuroendocrine tumours (GEP-NET), expressing somatostatin receptors in adults.*	*N.A.*
voretigene neparvovec	*Is indicated for the treatment of adult and paediatric patients with vision loss due to inherited retinal dystrophy caused by confirmed*	*Is indicated for the treatment of adults and children with visual loss due to hereditary retinal dystrophy resulting from confirmed bi-allelic*	*Is indicated for the treatment of adult and paediatric patients with vision loss due to inherited retinal dystrophy caused by confirmed*

	biallelic RPE65 mutations and who have sufficient viable retinal cells.	*mutations in the RPE65 gene and having sufficient viable retinal cells.*	*biallelic RPE65 mutations and who have sufficient viable retinal cells.*
olaparib	*In combination with bevacizumab is indicated for the maintenance treatment of adult patients with advanced high-grade epithelial ovarian cancer (stages III and IV according to FIGO), fallopian tube cancer, or primary peritoneal cancer, in response (complete or partial) after completion of first-line platinum-based chemotherapy in combination with bevacizumab and whose tumour has homologous recombination deficiency (HRD) without a BRCA1/2 mutation.*	*In combination with bevacizumab is indicated for the maintenance treatment of adult patients with advanced (FIGO stages III and IV) high-grade epithelial ovarian, fallopian tube or primary peritoneal cancer who are in response (complete or partial) following completion of first-line platinum-based chemotherapy in combination with bevacizumab and whose cancer is associated with homologous recombination deficiency (HRD) positive status defined by either a BRCA1/2 mutation and/or genomic instability.*	*Is indicated for the maintenance treatment of adult patients with advanced (FIGO stages III and IV) high-grade epithelial ovarian, fallopian tube or primary peritoneal cancer who are in response (complete or partial) following completion of first-line platinum-based chemotherapy in combination with bevacizumab and whose cancer is associated with homologous recombination deficiency HRD) positive status defined by either a BRCA1/2 mutation and/or genomic instability.*
olaparib	*Is indicated, as monotherapy, for the treatment of adult patients with metastatic castration-resistant prostate cancer with mutations in the BRCA1/2 genes (germline mutation and/or somatic mutation), progressing after previous treatment that included a new hormonal agent.*	*Is indicated, as monotherapy for the treatment of adult patients with metastatic castration-resistant prostate cancer and BRCA1/2-mutations (germline and/or somatic) who have progressed following prior therapy that included a new hormonal agent.*	*Is indicated, as monotherapy for the treatment of adult patients with metastatic castration-resistant prostate cancer and BRCA1/2-mutations (germline and/or somatic) who have progressed following prior treatment that included a new hormonal agent.*
olaparib	*Is indicated, as monotherapy for the maintenance treatment of adult patients with advanced (FIGO stages III and IV) BRCA1/2-mutated (germline and/or somatic) high-grade epithelial ovarian, fallopian tube or primary peritoneal cancer who are in response (complete or partial) following completion of first-line platinum-based chemotherapy.*	*Is indicated, as monotherapy for the maintenance treatment of adult patients with platinum-sensitive relapsed BRCA-mutated (germline and/or somatic) high-grade serous epithelial ovarian, fallopian tube or primary peritoneal cancer who are in response (complete or partial response) to platinum-based chemotherapy.*	*Is indicated, as monotherapy for the maintenance treatment of adult patients with advanced (FIGO stages III and IV) BRCA1/2-mutated (germline and/or somatic) high-grade epithelial ovarian, fallopian tube or primary peritoneal cancer who are in response (complete or partial) following completion of first-line platinum-based chemotherapy.*
olaparib	*Is indicated as monotherapy for the treatment of adult patients with germline BRCA1/2-mutations, who have HHER2-negativelocally advanced or metastatic breast*	*Is indicated for the treatment of HER2-negative locally advanced or metastatic breast cancer with*	*Is indicated as monotherapy for the treatment of adult patients with germline BRCA1/2 mutations, who have HER2-negative locally advanced or metastatic breast*

	cancer. Patients should have previously been treated with an anthracycline and a taxane in the (neo)adjuvant or metastatic setting unless patients were not suitable for these treatments.	*germline mutation of the BRCA1/2 genes.*	*cancer. Patients should have previously been treated with an anthracycline and a taxane in the (neo)adjuvant or metastatic setting unless patients were not suitable for these treatments.*
rituximab	*Is indicated for the treatment of patients with moderate to severe pemphigus vulgaris (PV).*	*Is indicated for the treatment of patients with moderate to severe pemphigus vulgaris.*	*N.A.*
cladribine	*Is indicated for the treatment of adult patients with high-activity relapsing remitting multiple sclerosis according to the following clinical or imaging characteristics: patients with 1 relapse in the previous year and at least 1 Gd+ lesion in T1 or 9 or more lesions in T2 during therapy with other DMDs; patients with 2 or more relapses in the previous year, whether or not treated with DMDs.*	*Is indicated for the treatment of adult patients with highly active relapsing multiple sclerosis (MS) as defined by clinical or imaging (MRI) features.*	*Is indicated for the treatment of adult patients with highly active relapsing multiple sclerosis (MS) as defined by clinical or imaging features.*
glecaprevir/pibrentasvir	*Is indicated for the treatment of chronic hepatitis C virus (HCV) infection in adults.*	*Is indicated for the treatment of chronic hepatitis C virus (HCV) infection in adults and adolescents aged 12 years to under 18 years.*	*Is indicated for the treatment of chronic hepatitis C virus (HCV) infection in adults.*
glecaprevir/pibrentasvir	*Is indicated for the treatment of chronic hepatitis C virus (HCV) infection in adults and adolescents aged 12 to <18 years (Therapeutic innovation about indication: treatment of chronic hepatitis C virus (HCV) infection in adolescents aged 12 to <18 years).*	*Is indicated for the treatment of chronic hepatitis C virus (HCV) infection in adults and adolescents aged 12 years to under 18 years.*	*Is indicated for the treatment of chronic hepatitis C virus (HCV) infection in adults and in adolescents aged 12 to <18 years.*
trametinib	*In combination with dabrafenib is indicated for the adjuvant treatment of adult patients with Stage III melanoma with a BRAF V600 mutation, following complete resection.*	*In combination with dabrafenib is indicated for the adjuvant treatment of adult patients with BRAF V600 mutation-positive stage III melanoma after complete resection.*	*In combination with dabrafenib is indicated for the adjuvant treatment of adult patients with Stage III melanoma with a BRAF V600 mutation, following complete resection.*
binimetinib	*In combination with encorafenib is indicated for the treatment of adult patients with unresectable or*	*In combination with encorafenib is indicated for the treatment of unresectable or metastatic*	*In combination with encorafenib is indicated for the treatment the treatment of adult patients with unresectable or metastatic*

	metastatic melanoma with a BRAF V600 mutation.	*melanoma in adults with a BRAF V600 mutation.*	*melanoma with a BRAF V600 mutation.*
vestronidase alfa	*Is indicated for the treatment of non-neurologic manifestations of mucopolysaccharidosis VII (MPS VII; Sly's syndrome).*	*N.A.*	*Is indicated for the treatment of non-neurologic manifestations of mucopolysaccharidosis VII (MPS VII; Sly's syndrome).*
tafasitamab	*Is indicated in combination with lenalidomide, followed by tafasitamab as monotherapy, for the treatment of adult patients with relapsed or refractory Diffuse Large B-Cell Lymphoma (DLBCL) who are ineligible for Autologous Stem Cell Transplant (ASCT).*	*Is indicated in combination with lenalidomide, followed by tafasitamab as monotherapy for the treatment of adult patients with diffuse large B-cell lymphoma only: - as 2nd line therapy in patients not eligible for hematopoietic stem cell autotransplantation (HSCT), - and in 3rd line and beyond, only in patients ineligible for tisagenlecleucel and axicabtagene ciloleucel.*	*Is indicated in combination with lenalidomide, followed by tafasitamab as monotherapy, for the treatment of adult patients with relapsed or refractory Diffuse Large B-Cell Lymphoma (DLBCL) who are ineligible for Autologous Stem Cell Transplant (ASCT).*
lusutrombopag	*Is indicated for the treatment of severe thrombocytopenia in adult patients with chronic liver disease undergoing invasive procedures.*	*N.A.*	*Is indicated for the treatment of severe thrombocytopenia in adult patients with chronic liver disease undergoing invasive procedures.*
metreleptin	*Is indicated as an adjunct to diet as a replacement therapy to treat the complications of leptin deficiency in lipodystrophy (LD) patients with confirmed congenital generalised LD (Berardinelli-Seip syndrome) or acquired generalised LD (Lawrence syndrome) in adults and children 2 years of age and above.*	*Is indicated in addition to diet as replacement therapy to treat complications associated with leptin deficiency in patients with lipodystrophy (LD): with confirmed congenital generalised LD (Berardinelli-Seip syndrome) or acquired LD (Lawrence syndrome) in patients >2 years of age; with confirmed familial partial LD or acquired LD (Barraquer-Simons syndrome) in patients >12 years of age in whom standard therapies have failed to achieve adequate metabolic control.*	*Is indicated as an adjunct to diet as a replacement therapy to treat the complications of leptin deficiency in lipodystrophy (LD) patients with confirmed congenital generalised LD (Berardinelli-Seip syndrome) or acquired generalised LD (Lawrence syndrome) in adults and children 2 years of age and above.*
obeticholic acid	*Is indicated for the treatment of primary biliary cholangitis (also known as primary biliary cirrhosis) in combination with ursodeoxycholic acid (UDCA) in adults with an inadequate response*	*Is indicated for the treatment of primary biliary cholangitis (also known as primary biliary cirrhosis) in combination with ursodeoxycholic acid (UDCA) in adults with an inadequate response*	*Is indicated for the treatment of primary biliary cholangitis (PBC) in combination with ursodeoxycholic acid (UDCA) in adults with an inadequate response*

	to UDCA or as monotherapy in adults unable to tolerate UDCA.	*to UDCA or as monotherapy in adults unable to tolerate UDCA.*	*to UDCA or as monotherapy in adults unable to tolerate UDCA.*
ocrelizumab	*Is indicated for the treatment of adult patients with early primary progressive multiple sclerosis (PPMS) in terms of disease duration and level of disability, and with imaging features characteristic of inflammatory activity.*	*Is indicated for the treatment of early-stage PP-MS in terms of duration of disease and degree of disability, associated with imaging data characteristic of inflammatory activity.*	*Is indicated for the treatment of adult patients with early primary progressive multiple sclerosis (PPMS) in terms of disease duration and level of disability, and with imaging features characteristic of inflammatory activity*
nintedanib	*Is indicated in adults for the treatment of systemic sclerosis-associated interstitial lung disease (SSc-ILD).*	*Is indicated in adults for the treatment of systemic sclerosis associated interstitial lung disease (SSc-ILD).*	*Is indicated in adults for the treatment of systemic sclerosis associated interstitial lung disease (SSc-ILD).*
baricitinib	*Is indicated for the treatment of moderate to severe active rheumatoid arthritis in adult patients who have responded inadequately to, or who are intolerant to one or more disease modifying anti rheumatic drugs. Baricitinib may be used as monotherapy or in combination with methotrexate.*	*Is indicated for the treatment of rheumatoid polyarthritis (RP) after failure of one or more disease-modifying treatments. It is a second-line treatment after failure of conventional disease-modifying treatments such as methotrexate (MTX) or a third-line treatment (failure of biotherapy) or beyond (failure of multiple conventional disease-modifying and/or biological treatments).*	*Is indicated for the treatment of moderate to severe active rheumatoid arthritis in adult patients who have responded inadequately to, or who are intolerant to one or more disease modifying anti rheumatic drugs. Baricitinib may be used as monotherapy or in combination with methotrexate.*
andexanet alfa	*Is indicated for adult patients treated with a direct factor Xa (FXa) inhibitor (apixaban or rivaroxaban) when reversal of anticoagulation therapy is required because of potentially fatal or uncontrolled bleeding.*	*N.A.*	*Is indicated for adult patients treated with a direct factor Xa (FXa) inhibitor (apixaban or rivaroxaban) when reversal of anticoagulation is needed due to life-threatening or uncontrolled bleeding.*
patisiran sodium	*Is indicated for the treatment of hereditary transthyretin-mediated amyloidosis (hATTR amyloidosis) in adult patients with stage 1 or stage 2 polyneuropathy.*	*Is indicated for the treatment of hereditary transthyretin amyloidosis (hATTR amyloidosis) in adults with stage 1 or stage 2 polyneuropathy.*	*Is indicated for the treatment of hereditary transthyretin-mediated amyloidosis (hATTR amyloidosis) in adult patients with stage 1 or stage 2 polyneuropathy.*
cenobamato	*Is indicated as adjunctive therapy of focal-onset seizures with or without secondary generalization in adult patients with epilepsy who have not been adequately controlled despite*	*Is indicated as adjunctive treatment of focal-onset seizures with or without secondary generalisation in adult patients with epilepsy who have not been adequately controlled*	*Is indicated for the adjunctive treatment of focal-onset seizures with or without secondary generalisation in adult patients with epilepsy who have not been adequately controlled despite a*

	a history of treatment with at least 2 antiepileptic drugs.	*despite treatment with at least two anti-epileptic medicinal products.*	*history of treatment with at least 2 anti-epileptic medicinal products.*
nivolumab	*As monotherapy is indicated for the treatment of head and neck squamous cell carcinoma in adults progressing during or after platinum-based therapy.*	*Is indicated for the treatment of adult patients with head and neck squamous cell cancer during or after platinum-based chemotherapy.*	*As monotherapy is indicated for the treatment of recurrent or metastatic squamous cell cancer of the head and neck in adults progressing on or after platinum-based therapy.*
nivolumab	*As monotherapy is indicated for the treatment of adult patients with relapsed or refractory classical Hodgkin lymphoma after autologous stem cell transplant (ASCT) and treatment with brentuximab vedotin.*	*Is indicated for the treatment of adults with relapsing or refractory classical Hodgkin lymphoma (cHL) after autologous haematopoieticstem cell transplant (ASCT) and treatment with brentuximab vedotin.*	*As monotherapy is indicated for the treatment of adult patients with relapsed or refractory classical Hodgkin lymphoma after autologous stem cell transplant (ASCT) and treatment with brentuximab vedotin.*
nivolumab	*Is indicated in combination with ipilimumab for the first-line treatment of adult patients with unresectable malignant mesothelioma of the pleura with nonepithelial histology.*	*Is indicated for the first-line treatment of adult patients with unresectable malignant pleural mesothelioma.*	*In combination with ipilimumab is indicated for the first-line treatment of adult patients with unresectable malignant pleural mesothelioma and non-epithelioid tumour histology.*
nivolumab	*As monotherapy is indicated for the treatment of locally advanced unresectable or metastatic urothelial carcinoma in adults after failure of previous platinum-based therapy.*	*N.A.*	*As monotherapy is indicated for the treatment of locally advanced unresectable or metastatic urothelial carcinoma in adults after failure of previous platinum-based therapy.*
nivolumab	*In combination with ipilimumab is indicated for first-line treatment of adult patients with intermediate/poor-risk advanced renal cell carcinoma.*	*Is indicated in combination with ipilimumab for the first-line treatment of adults with advanced renal cell carcinoma of intermediate/favourable prognosis.*	*In combination with ipilimumab is indicated for first-line treatment of adult patients with intermediate/poor-risk advanced renal cell carcinoma.*
nivolumab	*As monotherapy is indicated for the adjuvant treatment of adults with melanoma with involvement of lymph nodes or metastatic disease who have undergone complete resection.*	*As monotherapy is indicated for the adjuvant treatment of adults with melanoma with involvement of lymph nodes or metastatic disease who have undergone complete resection.*	*As monotherapy is indicated for the adjuvant treatment of adults with melanoma with involvement of lymph nodes or metastatic disease who have undergone complete resection.*
nivolumab	*With fluoropyrimidine- and platinum-based combination chemotherapy is indicated for the first-line treatment of adult patients with HER2-negative, advanced or*	*In combination with fluoropyrimidine and platinum-based chemotherapy is a first-line treatment for adult patients with advanced or metastatic*	*With fluoropyrimidine- and platinum-based combination chemotherapy is indicated for the first-line treatment of adult patients with HER2-negative advanced or*

	metastatic adenocarcinoma of the stomach, gastro-oesophageal junction, or esophagus whose tumors express PD-L1 with a combined positive score (CPS) ≥ 5.	*HER-2 negative gastric, oesophageal junction or oesophageal adenocarcinoma whose tumours express PD-L1 with a Combined Positive Score (CPS) ≥ 5.*	*metastatic gastric, gastro-oesophageal junction or oesophageal adenocarcinoma whose tumours express PD-L1 with a combined positive score (CPS) ≥ 5.*
cenegermin	*Is indicated for the treatment of moderate (persistent epithelial defect) or severe (corneal ulcer) neurotrophic keratitis in adults.*	*Is indicated for the treatment of adults with moderate (persistent epithelial lesions) or severe (corneal ulcer) neurotrophic keratitis.*	*Is indicated for the treatment of moderate (persistent epithelial defect) or severe (corneal ulcer) neurotrophic keratitis in adults.*
lumasiran	*Is indicated for the treatment of primary hyperoxaluria type 1 (PH1) in all age groups.*	*Is indicated for the treatment of primary hyperoxaluria type 1 (PH1) in all age groups.*	*Is indicated for the treatment of primary hyperoxaluria type 1 (PH1) in all age groups.*
pegvaliase	*Is indicated for the treatment of patients with phenylketonuria (PKU) aged 16 years and older who have inadequate blood phenylalanine control (blood phenylalanine levels greater than 600 micromol/l) despite prior management with available treatment options.*	*Is indicated for the treatment of patients with phenylketonuria (PKU) aged 16 years and older who have inadequate blood phenylalanine control (blood phenylalanine levels greater than 600 micromol/l) despite prior management with available treatment options.*	*Is indicated for the treatment of patients with phenylketonuria (PKU) aged 16 years and older who have inadequate blood phenylalanine control (blood phenylalanine levels greater than 600 µmol/l) despite prior management with available treatment options.*
pertuzumab	*Is indicated in combination with trastuzumab and chemotherapy in: neoadjuvant treatment of adult patients with HER2-positive, locally advanced, inflammatory or early-stage breast cancer at high risk of recurrence.*	*N.A.*	*Is indicated in combination with trastuzumab and chemotherapy in: neoadjuvant treatment of adult patients with HER2-positive, locally advanced, inflammatory or early-stage breast cancer at high risk of recurrence; adjuvant treatment of adult patients with HER2-positive early-stage breast cancer at high risk of recurrence.*
doravirina	*Is indicated, in combination with other antiretroviral medicinal products, for the treatment of adults infected with HIV 1 without past or present evidence of resistance to the NNRTI class.*	*Is indicated for the treatment of HIV-1 infection in patients with a low viral load ≤ 100,000 copies/mL, when a non-nucleoside reverse transcriptase inhibitor (NNRTI) is indicated and the use of rilpivirine is not appropriate, but there is no demonstrated clinical benefit in the management of these patients.*	*Is indicated, in combination with other anti-retroviral medicinal products, for the treatment of adults infected with the human immunodeficiency virus (HIV-1). The HI viruses must not have mutations known to be associated with resistance to the NNRTI (nonnucleosidic reverse*

			transcriptase inhibitor) class of substances.
polatuzumab vedotin	*In combination with bendamustine and rituximab is indicated for the treatment of adult patients with relapsed/refractory diffuse large B-cell lymphoma (DLBCL) who are not candidates for transplantation of hematopoietic stem cells.*	*N.A.*	*In combination with bendamustine and rituximab is indicated for the treatment of adult patients with relapsed/refractory diffuse large B-cell lymphoma (DLBCL) who are not candidates for haematopoietic stem cell transplant.*
mogamulizumab	*Is indicated for the treatment of adult patients with mycosis fungoides (MF) or Sézary syndrome (SS) who have received at least one prior systemic therapy.*	*Is indicated for the treatment of adults with mycosis fungoides or Sézary syndrome who have received at least one previous systemic treatment.*	*Is indicated for the treatment of adult patients with mycosis fungoides (MF) or Sézary syndrome (SS) who have received at least one prior systemic therapy.*
alirocumab	*Is indicated in adults with established atherosclerotic cardiovascular disease to reduce cardiovascular risk by lowering LDL-C levels, as an adjunct to correction of other risk factors:*<br>*-in combination with the maximum tolerated dose of a statin with or without other lipid-lowering therapies or,*<br>*-alone or in combination with other lipid-lowering therapies in patients who are statin-intolerant, or for whom a statin is contraindicated.*	*Is indicated in combination with optimised lipid-lowering therapy in adult patients with heterozygous familial hypercholesterolaemia, inadequately controlled and requiring treatment with LDL-apheresis; In combination with optimised lipid-lowering therapy in adult patients with established atherosclerotic cardiovascular disease with a history of recent ACS (secondary prevention) who are uncontrolled (LDL-c ≥ 0.7 g/L) despite optimised lipid-lowering therapy including at least one statin at the maximum tolerated dose.*	*Is indicated in adults with established atherosclerotic cardiovascular disease to reduce cardiovascular risk by lowering LDL-C levels, as an adjunct to correction of other risk factors: - in combination with the maximum tolerated dose of a statin with or without other lipid-lowering therapies or, - alone or in combination with other lipid-lowering therapies in patients who are statin-intolerant, or for whom a statin is contraindicated.*
letermovir	*Is indicated for prophylaxis of cytomegalovirus (CMV) reactivation and disease in adult CMV-seropositive recipients [R+] of an allogeneic haematopoietic stem cell transplant (HSCT).*	*Is indicated for prevention of cytomegalovirus (CMV) reactivation and CMV disease in adult CMV-seropositive allogeneic haematopoietic stem cell transplantation (HSCT) recipients [R+].*	*Is indicated for prophylaxis of cytomegalovirus (CMV) reactivation and disease in adult CMV-seropositive recipients [R+] of an allogeneic haematopoietic stem cell transplant (HSCT).*
dinutuximab beta	*Is indicated in the treatment of high-risk neuroblastoma in patients 12 months of age and older who have previously undergone induction chemotherapy achieving at least a partial response, followed*	*Is indicated in the treatment of patients >12 months of age with high-risk neuroblastoma who have previously received induction chemotherapy with at least a partial response, followed by myeloablative*	*N.A.*

	by myeloablative therapy and stem cell transplantation, as well as in patients with a clinical history of relapsed or refractory neuroblastoma, with or without residual disease. Before treatment of relapsed neuroblastoma, any disease in active progression should be stabilized by other appropriate measures.	*therapy and haematopoietic stem cell transplantation, and in patients with relapsed or refractory neuroblastoma, with or without residual disease.*	
dinutuximab beta	*Is indicated in patients with a clinical history of relapsed/refractory disease and in patients who have not achieved a complete response after first-line therapy, it should be combined with interleukin-2 (IL-2) therapy.*	*Is indicated in patients with a history of relapsed or refractory disease and in patients who have failed to respond to refractory disease and in patients who have not achieved a complete response it should be combined with interleukin 2 (IL-2).*	*N.A.*
imipenem/cilastatina/ relebactam	*Is indicated for the treatment of adult inpatients with infections, including HAP/VAP and associated bacteremia, caused by carbapenemase-resistant Gram-negative bacteria in whom there are limited therapeutic options or with invasive infections with a strongly suspected etiology from carbapenemase-resistant Gram-negative bacteria (as per AIFA prescription form).*	*Is indicated for the treatment of patients with enterobacterial infections sensitive to the imipenem/cilastatin/relebactam combination and for whom the use of other beta-lactams and carbapenems (meropenem or imipenem-cilastatin) is not feasible in case of resistance, in particular due to the production of KPC-type carbapenemases.*	*Is indicated for: - Treatment of hospital-acquired pneumonia (HAP), including ventilator-associated pneumonia (VAP) in adults. -Treatment of bacteraemia that occurs in association with, or is suspected to be associated with HAP or VAP, in adults.-Treatment of infections due to aerobic Gram-negative organisms in adults with limited treatment options. Consideration should be given to official guidelines on the appropriate use of antibiotics.*
selpercatinib	*As monotherapy is indicated in the treatment of adult patients with advanced RET fusion-positive non-small cell lung cancer (NSCLC) requiring systemic therapy after previous treatment with immunotherapy and/or platinum-based chemotherapy.*	*As monotherapy is indicated in: - the treatment of adult patients with advanced non-small cell lung cancer (NSCLC) with a RET gene fusion, who require systemic therapy after prior treatment with immunotherapy and/or platinum-based chemotherapy; - the treatment of adult and adolescent patients with advanced medullary thyroid cancer (MTC) with a RET gene mutation who require systemic*	*N.A.*

		therapy after prior treatment with cabozantinib and/or vandetanib.	
selpercatinib	*As monotherapy is indicated in the treatment of adults with advanced RET fusion-positive thyroid cancer requiring systemic therapy after previous treatment with sorafenib and/or lenvatinib.*	*As monotherapy is indicated in the treatment of adult patients with advanced RET-fused thyroid cancer who require systemic therapy after prior treatment with sorafenib and/or lenvatinib.*	*As monotherapy is indicated for the treatment of adults with advanced RET fusionpositive thyroid cancer who require systemic therapy following prior treatment with sorafenib and/or lenvatinib.*
selpercatinib	*As monotherapy is indicated for the treatment of adults and adolescents aged 12 years and older with advanced medullary thyroid carcinoma (MTC) with RET mutation requiring systemic therapy after previous treatment with cabozantinib and/or vandetanib.*	*As monotherapy is indicated in: - the treatment of adult patients with advanced non-small cell lung cancer (NSCLC) with a RET gene fusion, who require systemic therapy after prior treatment with immunotherapy and/or platinum-based chemotherapy; - the treatment of adult and adolescent patients with advanced medullary thyroid cancer (MTC) with a RET gene mutation who require systemic therapy after prior treatment with cabozantinib and/or vandetanib.*	*As monotherapy is indicated for the treatment of adults and adolescents 12 years and older with advanced RET-mutant medullary thyroid cancer (MTC) who require systemic therapy following prior treatment with cabozantinib and/or vandetanib.*
lenalidomide	*Is indicated as maintenance therapy of adult patients with newly diagnosed multiple myeloma undergoing autologous stem cell transplantation.*	*Is indicated as maintenance monotherapy for previously untreated multiple myeloma in adult patients who have received an autologous stem cell transplant.*	*N.A.*
lenalidomide	*In combination with rituximab (anti-CD20 antibody) is indicated for the treatment of adult patients with follicular lymphoma (grade 1-3a) previously treated.*	*Is indicated for the treatment of previously treated follicular lymphoma (grade 1, 2 or 3a) in adult patients who are non-refractory to rituximab (patients not previously treated with rituximab or who have not relapsed on treatment including rituximab or within 6 months of its discontinuation).*	*N.A.*
upadacitinib	*Is indicated for the treatment of moderate to severe active rheumatoid arthritis in adult patients who have responded inadequately to, or who are intolerant to one or more disease-modifying anti-rheumatic drugs*	*Is indicated for the treatment (as monotherapy or in combination with methotrexate) of moderate to severe active rheumatoid arthritis in adult patients who have had an inadequate response to, or*	*Is indicated for the treatment of moderate to severe active rheumatoid arthritis in adult patients who have responded inadequately to, or who are intolerant to one or more diseasemodifying anti-rheumatic*

	(DMARDs). It may be used as monotherapy or in combination with methotrexate.	*intolerance of, one or more background therapies.*	*drugs (DMARDs). It may be used as monotherapy or in combination with methotrexate.*
tocilizumab	*Is indicated for the treatment of giant cell arteritis (ACG) in adult patients.*	*Is indicated for the treatment of adults with giant cell arteritis (GCA).*	*N.A.*
entrectinib	*As monotherapy is indicated for the treatment of adult and pediatric patients aged 12 years and older with solid tumors expressing a neurotrophic receptor tyrosine kinase (NTRK) gene fusion, who have locally advanced, metastatic disease or whose surgical resection could result in severe morbidity and who have not been treated previously with an NTRK inhibitor, who have no satisfactory treatment options.*	*N.A.*	*As monotherapy is indicated for the treatment of adult and pediatric patients aged 12 years and older with solid tumors expressing a neurotrophic receptor tyrosine kinase (NTRK) gene fusion, who have locally advanced, metastatic disease or whose surgical resection could result in severe morbidity and who have not been treated previously with an NTRK inhibitor, who have no satisfactory treatment options.*
fostemsavir	*In combination with other antiretrovirals, is indicated for the treatment of adults with multidrug-resistant HIV-1 infection for whom a suppressive antiviral regimen cannot otherwise be established.*	*Is indicated for the treatment of adults with multi-drug resistant HIV-1 infection in whom it is otherwise impossible to establish a suppressive antiretroviral treatment regimen.*	*In combination with other antiretrovirals, is indicated for the treatment of adults with multidrug resistant HIV-1 infection for whom it is otherwise not possible to construct a suppressive anti-viral regimen.*
midostaurina	*In combination with standard induction chemotherapy with daunorubicin and cytarabine and consolidation chemotherapy with high-dose cytarabine is indicated for adult patients with newly diagnosed acute myeloid leukemia (AML) with FLT3 mutation positive.*	*Is indicated for the treatment of adults with newly diagnosed acute myeloid leukaemia (AML) with FLT3 mutation, in combination with standard induction chemotherapy with daunorubicin and cytarabine and consolidation chemotherapy with high-dose cytarabine, followed by maintenance treatment with midostaurina monotherapy for patients in complete remission.*	*In combination with standard induction chemotherapy with daunorubicin and cytarabine and consolidation chemotherapy with high-dose cytarabine is indicated for adult patients with newly diagnosed acute myeloid leukemia (AML) with FLT3 mutation positive.*
midostaurina	*As monotherapy is indicated for the treatment of adult patients with aggressive systemic mastocytosis (aggressive systemic mastocytosis, ASM), systemic mastocytosis associated with haematological*	*Is indicated for the treatment of adults with aggressive systemic mastocytosis (ASM), systemic mastocytosis associated with other haematological malignancies*	*As monotherapy is indicated for the treatment of adult patients with aggressive systemic mastocytosis (aggressive systemic mastocytosis, ASM), systemic mastocytosis associated with haematological*

	neoplasm (systemic mastocytosis with associated haematological neoplasm, SM-AHN), or mast cell leukemia (mast cell leukaemia, MCL).	*(SMAM), or mast cell leukaemia (MCL), as monotherapy.*	*neoplasm (systemic mastocytosis with associated haematological neoplasm, SM-AHN), or mast cell leukemia (mast cell leukaemia, MCL).*
isatuximab	*Is indicated in combination with carfilzomib and dexamethasone, for the treatment of adult patients with multiple myeloma who have received at least one and no more than 3 prior lines of therapy.*	*Is indicated in combination with carfilzomib and dexamethasone, for the treatment of adult patients with multiple myeloma who have received at least one prior therapy.*	*Is indicated in combination with carfilzomib and dexamethasone, for the treatment of adult patients with multiple myeloma who have received at least one prior therapy.*
isatuximab	*Is indicated in combination with pomalidomide and dexamethasone, for the treatment of adult patients with relapsed and refractory multiple myeloma (MM) who have received at least two prior therapies including lenalidomide and a proteasome inhibitor (PI) and have demonstrated disease progression on the last therapy.*	*Is indicated in combination with pomalidomide and dexamethasone, for the treatment of adult patients with relapsed and refractory multiple myeloma (MM) who have received at least two prior therapies including lenalidomide and a proteasome inhibitor (PI) and have demonstrated disease progression on the last therapy.*	*Is indicated in combination with pomalidomide and dexamethasone, for the treatment of adult patients with relapsed and refractory multiple myeloma who have received at least two prior therapies including lenalidomide and a proteasome inhibitor and have demonstrated disease progression on the last therapy.*
eculizumab	*Is indicated in the second-line treatment, after rituximab, of neuromyelitis optica spectrum disorder (NMOSD) in adult patients positive for anti-aquaporin 4 antibodies (AQP4) with a clinical history of at least one relapse in the past 12 months and an Expanded Disability Status Scale (EDSS) score ≤ 7.*	*Is indicated for the treatment of neuromyelitis optica spectrum disorder (NMOSD) in adult patients who are anti-aquaporin-4 (AQP4) antibody-positive with a relapsing course of the disease (2 relapses in the past year or 3 relapses in the past two years, including one in the past year), and not responding to background immunosuppressant therapy (rituximab, azathioprine, mycophenolate mofetil).*	*N.A.*
eculizumab	*Is indicated in the treatment of adults with refractory generalized Myasthenia gravis (MGg) in patients positive for anti-acetylcholine receptor (AChR) antibodies*	*N.A.*	*N.A.*
spheroids of human autologous matrix-associated chondrocytes	*Is indicated for repair of symptomatic articular cartilage defects of the femoral condyle and knee cap (grade III or IV,*	*N.A.*	*N.A.*

according to the defect size	*International Cartilage Regeneration & Joint Preservation Society [International tilage Regeneration & Joint Preservation Society, ICRS]) with defect sizes ≥ 2 cm2 and up to 10 cm2 in adults.*		
nusinersen	*Is indicated for the treatment of 5q Spinal Muscular Atrophy.*	*Is indicated for the etiological treatment of 5q spinal muscular atrophy.*	*Is indicated for the treatment of 5q spinal muscular atrophy.*
esketamina	*In combination with an SSRI or SNRI, is indicated for adults with treatment-resistant major depressive disorder who have not responded to at least two different antidepressant treatments during the current moderate to severe depressive episode.*	*In combination with an SSRI or SNRI, is indicated for adult patients under 65 years of age for the treatment of resistant major depressive episodes who have not responded to at least two different antidepressants from two different classes during the current severe depressive episode, and in cases where electroconvulsive therapy is contraindicated or resistant, or for patients who do not have access to it or have refused it.*	*In combination with a SSRI or SNRI, is indicated for adults with treatment-resistant Major Depressive Disorder, who have not responded to at least two different treatments with antidepressants in the current moderate to severe depressive episode.*
regorafenib	*Is indicated as monotherapy for the treatment of adult patients with hepatocellular carcinoma (HCC) who have been previously treated with sorafenib.*	*Is indicated as monotherapy in the treatment of adult patients with hepatocellular carcinoma (HCC) who have been treated with sorafenib.*	*N.A.*
dabrafenib	*In combination with trametinib is indicated for the adjuvant treatment of adult patients with Stage III melanoma with a BRAF V600 mutation, following complete resection.*	*Is indicated as adjuvant treatment of adult patients with BRAF V600 mutation-positive stage III melanoma after complete resection.*	*In combination with trametinib is indicated for the adjuvant treatment of adult patients with Stage III melanoma with a BRAF V600 mutation, following complete resection.*
osimertinib	*As monotherapy is indicated for adjuvant treatment after complete tumor resection in adult patients with stage IB-IIIA non-small cell lung cancer (NSCLC) whose tumor has exon 19 deletion or exon 21 (L858R) epidermal growth factor receptor (EGFR) substitution mutation.*	*Is indicated as adjuvant treatment after complete tumour resection and after adjuvant chemotherapy if indicated in adult patients with stage IB-IIIA non-small cell lung cancer (NSCLC) whose tumours have epidermal growth factor receptor (EGFR) exon 19 deletions or exon 21 (L858R) substitution mutations.*	*As monotherapy is indicated for the adjuvant treatment after complete tumour resection in adult patients with stage IB-IIIA non-small cell lung cancer (NSCLC) whose tumours have epidermal growth factor receptor (EGFR) exon 19 deletions or exon 21 (L858R) substitution mutations.*

osimertinib	*As monotherapy is indicated for the first-line treatment of adult patients with locally advanced or metastatic NSCLC with activating EGFR mutations.*	*As monotherapy is indicated for the first-line treatment of adult patients with locally advanced or metastatic non-small cell lung cancer (NSCLC) with activating epidermal growth factor receptor (EGFR) mutations.*	*As monotherapy is indicated for the first-line treatment of adult patients with locally advanced or metastatic NSCLC with activating EGFR mutations.*
lanadelumab	*Is indicated for routine prevention of recurrent attacks of hereditary angioedema (HAE) in patients aged 12 years and older.*	*Is indicated for prevention of recurrent attacks of hereditary angioedema (HAE) in patients aged 12 years and over.*	*Is indicated for routine prevention of recurrent attacks of hereditary angioedema (HAE) in patients aged 12 years and older.*
brexucabtagene autoleucel	*Is indicated for the treatment of adult patients with relapsed or refractory mantle cell lymphoma (MCL) after two or more lines of systemic therapy including a Bruton's tyrosine kinase (BTK) inhibitor.*	*Is indicated for the treatment of adult patients with relapsed or refractory mantle cell lymphoma after at least two lines of systemic therapy including treatment with a Bruton tyrosine kinase inhibitor.*	*Is indicated for the treatment of adult patients with relapsed or refractory mantle cell lymphoma (MCL) after two or more lines of systemic therapy including a Bruton's tyrosine kinase (BTK) inhibitor.*
atezolizumab	*As monotherapy is indicated for the treatment of adult patients with locally advanced or metastatic NSCLC after prior chemotherapy.*	*As monotherapy is indicated for first-line treatment of adult patients with metastatic non-small cell lung cancer (NSCLC) whose tumours have PD-L1 expression ≥ 50% on tumour cells (TC) or ≥ 10% on tumour-infiltrating immune cells (IC) and who do not have EGFR-mutated or ALK-rearranged (ALK-positive) NSCLC.*	*As monotherapy is indicated for the treatment of adult patients with locally advanced or metastatic NSCLC after prior chemotherapy. Patients with EGFR mutant or ALK-positive NSCLC should also have received targeted therapies before receiving atezolizumab.*
atezolizumab	*In combination with bevacizumab, paclitaxel, and carboplatin, is indicated for the first-line treatment of adult patients with metastatic non-squamous non-small cell lung cancer (NSCLC). In NSCLC patients with epidermal growth factor receptor (EGFR) or anaplastic lymphoma kinase (ALK)-positive mutations, atezolizumab, in combination with bevacizumab, paclitaxel, and carboplatin, is indicated only after failure of appropriate molecularly targeted therapies.*	*In combination with bevacizumab, paclitaxel, and carboplatin, is indicated for the first-line treatment of adult patients with metastatic non-squamous non-small cell lung cancer (NSCLC). In patients with EGFR mutation or ALK rearrangement, atezolizumab in combination with bevacizumab and relevant chemotherapy is indicated only after failure of appropriate targeted therapies.*	*In combination with bevacizumab, paclitaxel, and carboplatin, is indicated for the first-line treatment of adult patients with metastatic non-squamous non-small cell lung cancer (NSCLC). In patients with EGFR mutant or ALK-positive NSCLC, atezolizumab, in combination with bevacizumab, paclitaxel, and carboplatin, is indicated only after failure of appropriate targeted therapies.*

atezolizumab	*In combination with bevacizumab, is indicated for the treatment of adult patients with advanced or unresectable hepatocellular carcinoma (HCC) who have not received previous systemic therapy.*	*In combination with bevacizumab is indicated for the treatment of adult patients with advanced or unresectable hepatocellular carcinoma (HCC) who have not received prior systemic therapy only in patients with preserved liver function (Child-Pugh stage A), ECOG score 0 or 1, and who are ineligible for, or have failed, locoregional therapy.*	*In combination with bevacizumab, is indicated for the treatment of adult patients with advanced or unresectable hepatocellular carcinoma (HCC) who have not received prior systemic therapy.*
atezolizumab	*In combination with carboplatin and etoposide, is indicated for the first-line treatment of adult patients with extensive-stage small cell lung cancer (ES-SCLC).*	*Is indicated for the treatment of adult patients with extensive stage small cell lung cancer (SCLC) in first line, in combination with carboplatin and etoposide.*	*In combination with carboplatin and etoposide, is indicated for the first-line treatment of adult patients with extensive-stage small cell lung cancer (ES-SCLC).*
atezolizumab	*In combination with nab-paclitaxel is indicated for the treatment of adult patients with unresectable locally advanced or metastatic triple-negative breast cancer (TNBC) whose tumours have PD-L1 expression ≥ 1% and who have not received prior chemotherapy for metastatic disease.*	*Is indicated in combination with nab-paclitaxel for the treatment of unresectable or metastatic locally advanced triple-negative breast cancer whose tumours have PD-L1 expression ≥ 1% and have not previously received metastatic chemotherapy.*	*In combination with nab-paclitaxel is indicated for the treatment of adult patients with unresectable locally advanced or metastatic triple-negative breast cancer (TNBC) whose tumours have PD-L1 expression ≥ 1% and who have not received prior chemotherapy for metastatic disease.*
treosulfan	*In combination with fludarabine is indicated as part of a conditioning regimen, prior to allogeneic hematopoietic stem cell transplantation (alloHSCT), in adult patients with malignant and nonmalignant disease and in pediatric patients older than one month with malignant disease.*	*In combination with fludarabine,is indicated as a conditioning treatment prior to allogeneic haematopoietic stem cell transplantation (HSCT) in adult patients and in children aged over 1 month and adolescents with malignant diseases. In combination with fludarabine as a conditioning treatment prior to allogeneic HSC transplantation in adult patients with non-malignant diseases.*	*N.A.*
sacituzumab govitecan	*As monotherapy is indicated for the treatment of adult patients with metastatic triple-negative or unresectable breast cancer (metastatic triple-negative breast cancer, mTNBC) who have previously received at least two*	*As monotherapy is indicated for the treatment of adult patients with unresectable or metastatic triple-negative breast cancer (TNBC) who have received at least two prior*	*As monotherapy is indicated for the treatment of adult patients with unresectable or metastatic triple-negative breast cancer (mTNBC) who have received two or more prior systemic therapies, including*

	systemic therapies, at least one of which was for advanced disease.	*systemic treatments, including at least one for advanced disease.*	*at least one of them for advanced disease.*
ibalizumab	*In combination with one or more other antiretrovirals, is indicated for the treatment of adults with drug-resistant human immunodeficiency virus (HIV-1) infection for whom a suppressive antiviral regimen would not otherwise be arranged.*	*Is indicated in the treatment of adults with multi-drug resistant HIV-1 infection in whom it is otherwise impossible to establish a suppressive antiretroviral treatment regimen.*	*In combination with other antiretroviral(s), is indicated for the treatment of adults infected with multidrug resistant HIV-1 infection for whom it is otherwise not possible to construct a suppressive antiviral regimen.*
tucatinib	*Is indicated in combination with trastuzumab and capecitabine for the treatment of adult patients with locally advanced or metastatic HER2-positive breast cancer who have received at least 2 previous anti-HER2 treatment regimens.*	*Is indicated in combination with trastuzumab and capecitabine, for the treatment of adult patients with HER2-positive locally advanced or metastatic breast cancer who have previously received at least 2 anti-HER2 therapies.*	*Is indicated in combination with trastuzumab and capecitabine for the treatment of adult patients with locally advanced or metastatic HER2-positive breast cancer who have received at least 2 previous anti-HER2 treatment regimens.*
meropenem/vaborbactam	*Is indicated for the treatment of adult patients with established or suspected serious infections sustained by carbapenemase-resistant Enterobacteriaceae (CRE): established complicated urinary tract infection (cUTI), including pyelonephritis; established or suspected complicated intra-abdominal infection (cIAI); established or suspected nosocomial pneumonia (HAP), including ventilator-associated pneumonia (VAP); bacteremia occurring in association or suspected association with any of the infections listed above; treatment of established infections due to aerobic Gram-negative organisms in adults with limited treatment options.*	*Is indicated as a last resort for the treatment of patients with enterobacterial infections sensitive to the meropenem/vaborbactam combination and for whom the use of other beta-lactams and carbapenems (meropenem or imipenem-cilastatin) is not feasible in the event of resistance, in particular through the production of KPC-type carbapenemases.*	*N.A.*
venetoclax	*In combination with rituximab is indicated for the treatment of adult patients with CLL who have received at least one prior therapy.*	*In combination with rituximab is indicated for the treatment of adult patients with chronic lymphocytic leukaemia (CLL) who have received at least one prior therapy.*	*In combination with rituximab is indicated for the treatment of adult patients with chronic lymphocytic leukaemia (CLL) who have received at least one prior therapy.*

venetoclax	*In combination with obinutuzumab is indicated for the treatment of adult patients with previously untreated chronic lymphatic leukemia (CLL) who are not candidates for FCR-type immunochemotherapy.*	*In combination with obinutuzumab is indicated for the treatment of previously untreated chronic lymphocytic leukaemia (CLL) patients with 17p deletion and/or TP53 mutation only or in patients without 17p deletion or TP53 mutation who are ineligible for fludarabine-based therapy.*	*In combination with obinutuzumab is indicated for the treatment of adult patients with previously untreated chronic lymphocytic leukaemia (CLL).*
abemaciclib	*Is indicated for the treatment of postmenopausal women with locally advanced or metastatic hormone receptor (HR)-negative human epidermal growth factor receptor type 2 (HER2)-positive breast cancer in combination with an aromatase inhibitor as initial endocrine therapy.*	*Is indicated for the treatment of women with advanced breast cancer, HR+/HER2 - in combination with an aromatase inhibitor or fulvestrant as first-line hormone therapy, or in women previously treated with hormone therapy. In pre/perimenopausal women, hormone therapy should be combined with an LHRH agonist.*	*Is indicated for the treatment of postmenopausal women with hormone receptor (HR) positive, HER2-negative locally advanced or metastatic breast cancer who have not yet received initial endocrine therapy have received.*
abemaciclib	*Is indicated for the treatment of women with hormone receptor (HR) positive, human epidermal growth factor receptor 2 (HER2) negative locally advanced or metastatic breast cancer in combination with fulvestrant.*	*Is indicated in combination with fulvestrant, for the treatment of postmenopausal women with locally advanced or metastatic HR+/HER2- breast cancer, without symptomatic, life-threatening visceral involvement in the short term, in first-line metastatic women in early relapse from adjuvant hormonal therapy and in second-line metastatic women after a first line of hormonal therapy.*	*Is indicated is indicated for the treatment of women with hormone receptor (HR) positive, human epidermal growth factor receptor 2 (HER2) negative locally advanced or metastatic breast cancer in combination with an aromatase inhibitor or fulvestrant as initial endocrine-based therapy, or in women who have received prior endocrine therapy. In pre- or perimenopausal women, the endocrine therapy should be combined with a LHRH agonist.*
larotrectinib solfato	*As monotherapy is indicated for the treatment of adult and paediatric patients with solid tumours that display a Neurotrophic Tyrosine Receptor Kinase (NTRK) gene fusion,*<br>*- who have a disease that is locally advanced, metastatic or where surgical resection is likely to result in severe morbidity, and*	*Is indicated for the treatment of paediatrics and adults in all solid tumours with a fusion of the NTRK (Neurotrophic Tyrosine Receptor Kinase) gene: -locally advanced or metastatic, or where surgical resection would be likely to result in severe morbidity, and*<br>*-when there is no satisfactory treatment option.*	*As monotherapy is indicated for the treatment of adult and paediatric patients with solid tumours that display a Neurotrophic Tyrosine Receptor Kinase (NTRK) gene fusion, - who have a disease that is locally advanced, metastatic or where surgical resection is likely to result in severe morbidity, and - who have no satisfactory treatment options.*

	- who have no satisfactory treatment options.		
sofosbuvir/velpatasvir/ voxilaprevir	*Is indicated for the treatment of chronic hepatitis C virus (HCV) infection in adults.*	*Is indicated for the treatment of chronic hepatitis C virus (HCV) infection in adults.*	*Is indicated for the treatment of chronic hepatitis C virus (HCV) infection in adults.*
vosoritide	*Is indicated in the treatment of achondroplasia in patients aged 5 to 14 years at the time of initiation of therapy and whose epiphyses are not closed. The diagnosis of achondroplasia must be confirmed by appropriate genetic analysis.*	*Is indicated in the treatment of achondroplasia in patients aged 2 years and older whose epiphyses are not fused. The diagnosis of achondroplasia should be confirmed by appropriate genetic screening.*	*Is indicated for the treatment of achondroplasia in patients 2 years of age and older whose epiphyses are not closed. The diagnosis of achondroplasia should be confirmed by appropriate genetic testing.*
tafamidis	*Is indicated for the treatment of wild type or inherited transthyretin amyloidosis in adult patients with cardiomyopathy (ATTR-CM) in NYHA class I and II.*	*Is indicated for the treatment of wild-type or inherited transthyretin amyloidosis in adult patients with cardiomyopathy (ATTR-CM).*	*Is indicated for the treatment of wild-type or hereditary transthyretin amyloidosis in adult patients with cardiomyopathy (ATTR-CM).*
daunorubicin hydrochloride, cytarabine	*Is indicated for the treatment of adults with newly diagnosed, therapy-related acute myeloid leukaemia (t-AML) or AML with myelodysplasia-related changes (AML-MRC).*	*Is indicated for the treatment of adults with newly diagnosed, therapy-related acute myeloid leukaemia (t-AML) or AML with myelodysplasia-related changes (AML-MRC).*	*Is indicated for the treatment of adults with newly diagnosed, therapy-related acute myeloid leukaemia (t-AML) or AML with myelodysplasia-related changes (AML-MRC).*
volanesorsen	*Is indicated as an adjunct to diet in adult patients with genetically confirmed familial chylomicronemia syndrome (FCS) and at high risk for pancreatitis, in whom response to diet and triglyceride lowering therapy has been inadequate. It is reimbursed in adult patients with genetically confirmed familial chylomicronemia syndrome (FCS) and a history of acute pancreatitis (at least 1 episode in the past 5 years) despite diet and triglyceride-lowering therapy with fibrates and omega-3 fatty acids.*	*Is indicated as an adjunct to diet in adult patients with genetically confirmed familial chylomicronemia syndrome (FCS) and at high risk for pancreatitis, in whom response to diet and triglyceride lowering therapy has been inadequate.*	*Is indicated as an adjunct to diet in adult patients with genetically confirmed familial chylomicronemia syndrome (FCS) and at high risk for pancreatitis, in whom response to diet and triglyceride lowering therapy has been inadequate.*
rivaroxaban	*Administered together with acetylsalicylic acid (ASA), is indicated for the prevention of atherothrombotic events in adult*	*Is indicated for the prevention of atherothrombotic events, in combination with acetylsalicylic acid, only in adult patients with*	*N.A.*

	patients, at high risk of ischemic events, who have symptomatic coronary artery disease (CAD) or peripheral artery disease (PAD).	*severe peripheral arterial disease (PAD) who have recently undergone a successful surgical or endovascular lower limb revascularisation procedure and in whom rivaroxaban 2.5 mg is initiated within 10 days of revascularisation.*	
telotristat etiprate	*Is indicated for the treatment of carcinoid syndrome diarrhoea in combination with somatostatin analogue (SSA) therapy in adults inadequately controlled by SSA therapy.*	*Is indicated for the treatment of diarrhoea in carcinoid syndrome in combination with a somatostatin analogue (SSA) in adults, in case of insufficient control with SSA therapy.*	*Is indicated for the treatment of carcinoid syndrome diarrhoea in combination with somatostatin analogue (SSA) therapy in adults inadequately controlled by SSA therapy.*
gilteritinib fumarate	*Is indicated as monotherapy for the treatment of adult patients with relapsed or refractory acute myeloid leukemia (AML) with an FLT3 gene mutation and a nonfavorable cytogenetic risk class. "Maintenance" therapy post allogeneic hematopoietic stem cell transplantation (HSCT) is not eligible for reimbursement.*	*Is indicated for the treatment of adults with relapsed or refractory acute myeloid leukaemia (AML) with a FLT3 gene mutation.*	*Is indicated as monotherapy for the treatment of adult patients who have relapsed or refractory acute myeloid leukaemia (AML) with a FLT3 mutation.*
ipilimumab	*In combination with nivolumab is indicated for the first-line treatment of adult patients with intermediate/poor-risk advanced renal cell carcinoma.*	*Is indicated in the first-line treatment of adults with advanced renal cell carcinoma of intermediate/favourable prognosis.*	*In combination with nivolumab is indicated for first-line treatment of adult patients with intermediate/poor-risk advanced renal cell carcinoma.*
ipilimumab	*In combination with nivolumab for the first-line treatment of adult patients with unresectable malignant mesothelioma of the pleura with nonepithelial histology.*	*Is indicated in the first-line treatment of adult patients with unresectable malignant pleural mesothelioma.*	*N.A.*
axicabtagene ciloleucel	*Is indicated for the treatment of adult patients with relapsed or refractory diffuse large B cell lymphoma (DLBCL) and primary mediastinal large B cell lymphoma (PMBCL), after two or more lines of systemic therapy.*	*Is for the treatment of adult patients with diffuse large B-cell lymphoma and primary mediastinal large B-cell lymphoma who are refractory or relapsed after the second or subsequent line of systemic therapy.*	*Is indicated for the treatment of adult patients with relapsed or refractory diffuse large B cell lymphoma (DLBCL) and primary mediastinal large B cell lymphoma (PMBCL), after two or more lines of systemic therapy.*
linfociti T allogenici	*Is indicated as an adjunctive treatment in haploidentical*	*N.A.*	*Is indicated as an adjunctive treatment in haploidentical*

	hematopoietic stem cell transplantation (HSCT) of adult patients with high-risk hematologic malignancies.		*hematopoietic stem cell transplantation (HSCT) of adult patients with high-risk hematologic malignancies.*
ceftazidime/avibactam	*Is indicated for the treatment of the following infections in adults:*<br>*-complicated intra-abdominal infection (cIAI)*<br>*- complicated urinary tract infection (cUTI), including pyelonephritis*<br>*-hospital-acquired pneumonia (HAP), including pneumonia associated with mechanical ventilation (VAP)*<br>*Treatment of infections caused by aerobic Gram-negative microorganisms in adult patients in whom there are limited therapeutic options.*	*Is indicated as a last resort for the treatment of patients with enterobacterial infections sensitive to the ceftazidime/avibactam combination and for whom the use of other beta-lactams and carbapenems (meropenem or imipenem-cilastatin) cannot be envisaged in the event of resistance, in particular due to the production of KPC or OXA-48 type carbapenemases*	*Is indicated in adults and paediatric patients aged 3 months and older for the treatment of the following infections :*<br>*-Complicated intra-abdominal infection (cIAI)*<br>*- Complicated urinary tract infection (cUTI), including pyelonephritis*<br>*-Hospital-acquired pneumonia (HAP), including ventilator associated pneumonia (VAP)*<br>*Treatment of adult patients with bacteraemia that occurs in association with, or is suspected to be associated with, any of the infections listed above.*<br>*It is also indicated for the treatment of infections due to aerobic Gram-negative organisms in adults and paediatric patients aged 3 months and older with limited treatment options.*
niraparib	*Is indicated as monotherapy for the maintenance treatment of adult patients with platinum sensitive relapsed high grade serous epithelial ovarian, fallopian tube, or primary peritoneal cancer who are in response (complete or partial) to platinum based chemotherapy.*	*Is indicated as maintenance treatment of patients with recurrent platinum-sensitive high-grade serous epithelial ovarian, fallopian tube or primary peritoneal cancer who are responding (complete or partial response) to platinum-based chemotherapy.*	*Is indicated as monotherapy for the maintenance treatment of adult patients with platinum-sensitive relapsed high grade serous epithelial ovarian, fallopian tube, or primary peritoneal cancer who are in response (complete or partial) to platinum-based chemotherapy.*
niraparib	*Is indicated as monotherapy for the maintenance treatment of adult patients with advanced epithelial ovarian carcinoma (FIGO stage III and IV), fallopian tube carcinoma, or primary, high-grade peritoneal*	*Is indicated as a single agent for the maintenance treatment of adult patients with advanced (FIGO stages III and IV) high-grade epithelial ovarian, fallopian tube or primary peritoneal cancer who are*	*Is used as monotherapy for maintenance treatment in adult patients with advanced epithelial (FIGO stages III and IV) high-grade carcinoma of the ovaries, fallopian tubes or with primary*

	carcinoma who are responding (complete or partial response) to first-line platinum-based chemotherapy.	*responding (complete or partial response) to a first line of platinum-based chemotherapy.*	*peritoneal carcinoma who have a response (complete or partial) after first-line platinum-based chemotherapy.*
ceftolozano/tazobactam	*Is indicated for the treatment of Hospital Acquired Pneumonia (HAP), including pneumonia associated with mechanical ventilation (VAP).*	*N.A.*	*Is indicated for the treatment of adults with hospital-acquired pneumonia, including ventilator-associated pneumonia.*
bezlotoxumab	*Is indicated for the prevention of recurrence of Clostridium difficile infection (CDI) in adults at high risk for recurrence of CDI.*	*Is indicated for the prevention of recurrent Clostridium difficile infection (CDI) in adults at high risk of CDI recurrence.*	*Is indicated for the prevention of recurrence of Clostridium difficile infection (CDI) in adults at high risk for recurrence of CDI.*
onasemnogene abeparvovec	*Is indicated for the treatment of spinal muscular atrophy (SMA) 5q in patients weighing up to 13.5 kg: clinical diagnosis of SMA type 1 and onset in the first six months of life, or genetic diagnosis of SMA type 1 (biallelic mutation in the SMN1 gene and up to two copies of the SMN2 gene).*	*Is indicated for the treatment of patients with 5q spinal muscular atrophy (biallelic mutation of the SMN1 gene), with a clinical diagnosis of SMA type 1 and 2 or pre-symptomatic, with up to 3 copies of the SMN2 gene.*	*Is indicated for the treatment of: - patients with 5q spinal muscular atrophy (SMA) with a biallelic mutation in the SMN1 gene and a clinical diagnosis of SMA Type 1, or - patients with 5q SMA with a biallelic mutation in the SMN1 gene and up to 3 copies of the SMN2 gene.*
abiraterone acetato	*Is indicated together with prednisone or prednisolone for the treatment of high-risk and newly diagnosed metastatic hormone-sensitive prostate cancer (mHSPC) in combination with androgen deprivation therapy (ADT) in adult men.*	*Is indicated in combination with prednisone or prednisolone for the treatment of newly diagnosed, high-risk, hormone-sensitive metastatic prostate cancer in adults in combination with androgen suppression therapy (ADT).*	*Is indicated together with prednisone or prednisolone for the treatment of high-risk and newly diagnosed metastatic hormone-sensitive prostate cancer (mHSPC) in combination with androgen deprivation therapy (ADT) in adult men.*

AIFA indicates Agenzia Italiana del Farmaco; API, Active pharmaceutical ingredient; G-BA, Gemeinsame Bundesausschuss; HAS, Haute Autorité de Santé; HTA, Health Technology Assessment.

Supplementary Figure 1. Concordance on ATV assessments among HTA bodies – Subgroup analysis.

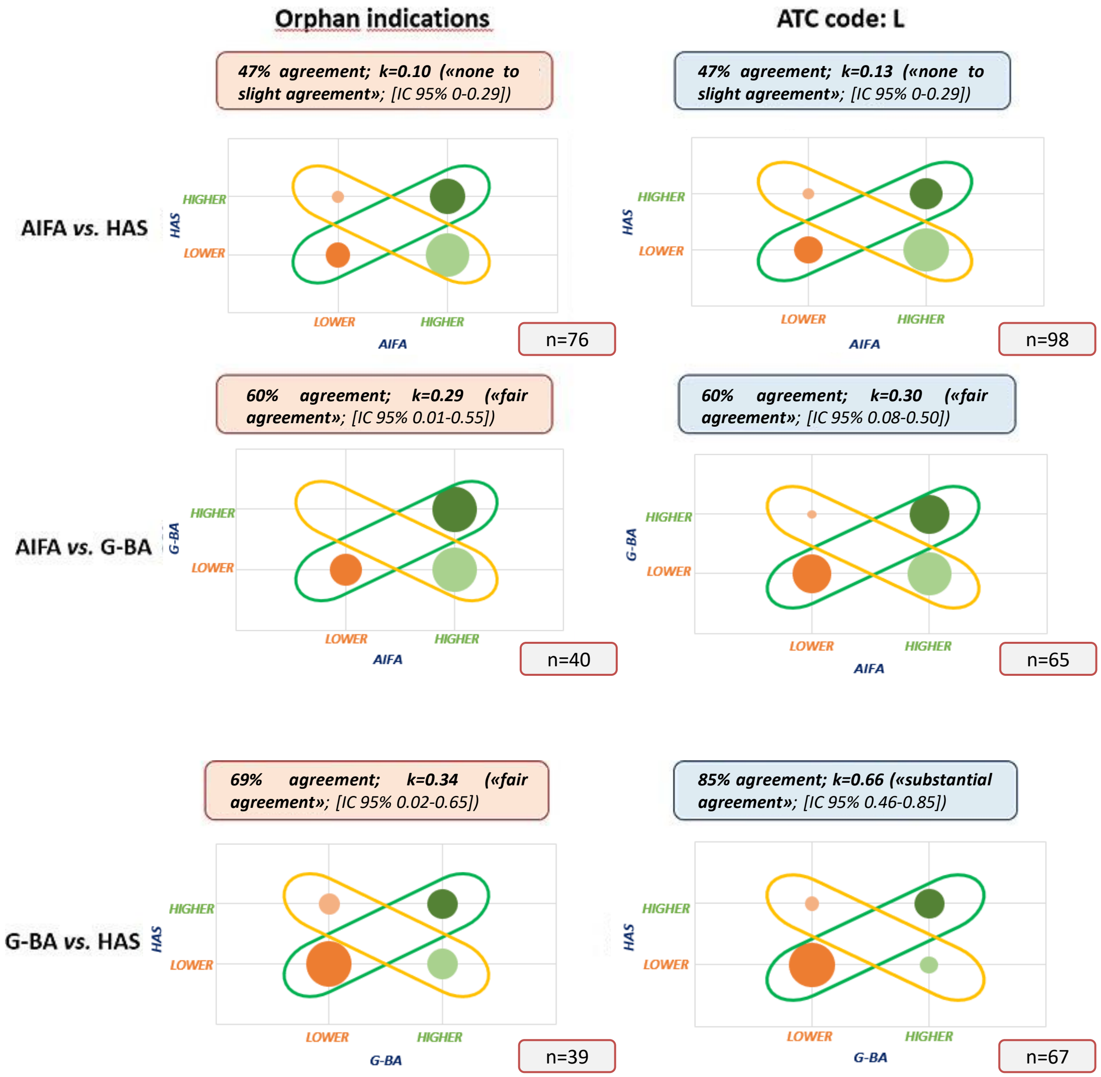

Note: the size of the bubbles represents the sample size

AIFA indicates Agenzia Italiana del Farmaco; ASMR, Amélioration du Service Médical Rendu; ATC, Anatomical Therapeutic Chemical Classification System; ATV, Added Therapeutic Value; CAB, Clinical Added Benefit; G-BA, Gemeinsame Bundesausschuss; HAS, Haute Autorité de Santé; HTA, Health Technology Assessment; L, Antineoplastic and immunomodulating agents.
